# Glycolytic and lipid oxidative metabolic programs are essential for freshly-isolated regulatory T cells in mice with sepsis†

**DOI:** 10.1039/d0ra01947j

**Published:** 2020-06-03

**Authors:** Xiaomei Zhu, WenQing Ji, Shubin Guo, Di Zhu, Yue Yang, Xin Liu

**Affiliations:** Beijing Key Laboratory of Cardiopulmonary-Cerebral Resuscitation, Emergency Department, Chao-Yang Hospital, Capital Medical University Chaoyang District Beijing 100020 China shubinguo@126.com

## Abstract

Immunometabolism determines the fate and function of regulatory T cells. The metabolic phenotype of regulatory T cells (Treg) is affected by various factors. The relationship between Treg metabolism and function of mice with sepsis is not clear. We used liquid chromatography and tandem mass spectrometry (LC-MS/MS) to analyze the metabolic profiles of freshly-isolated spleen Treg cells in mice with sepsis. It was found that in severe infection, activated Treg cells depend on glycolysis and fatty acid oxidation, and inhibition of metabolic pathways has a significant impact on the number and quality of Treg cells. Understanding the metabolic characteristics of Treg cells in the real environment in the body helps to grasp the function of Treg cells and even the overall immune status. Targeting the metabolic pathway of Treg may provide a new method for the treatment of sepsis.

## Abstract 2

Freshly-isolated Treg cells showed metabolic reprogramming in mice with sepsis, mainly manifested by increased glycolysis and fatty acid oxidation pathways.

## Graphical abstract

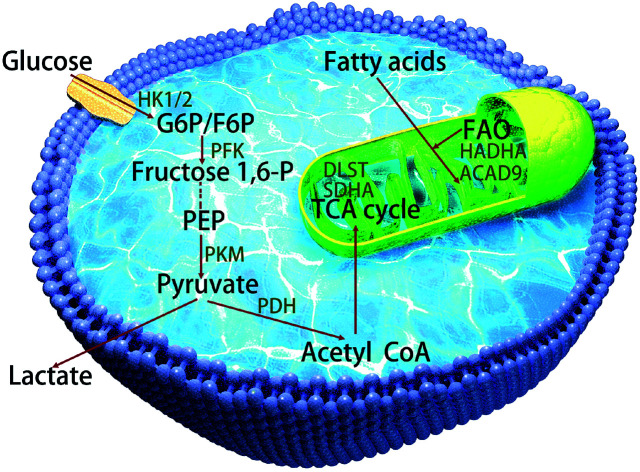

## 1. Introduction

Sepsis is a disease caused by an excessive immune response against the pathogen. Many studies have shown that hyperactivated innate immunity and immune-suppression occur simultaneously in sepsis. This immune disorder usually results in poor prognosis.^1–3^ Patients who survive an episode of sepsis often develop a long-lasting stage of immunosuppression making them susceptible to certain secondary infections.^4–6^ This immunosuppression is usually characterized by lymphopenia (T cell decline is most pronounced) and loss of immune function.^7,8^ The regulatory T cells are major immunoregulatory cells. Foxp3+ Treg cells limited the hyper-inflammatory response during early phase of sepsis,^9^ and induced immune-suppression in the later stages of sepsis.^10^ Under inflammatory conditions, activated immune cells turn metabolic reprogramming.^11–14^ Metabolic reconfiguration influences both the effector phase of inflammation and the resolution of inflammation by modulating immune cell fate and function. The factors controlling immunometabolism among different T cell subsets can also impact immune phenotype.^15^ Immunometabolism determines the fate of T cells and their immunological function under various pathological conditions including cancers, infections and autoimmune diseases.^16–20^ Therefore, studying the metabolism and function of immune cells under severe infection will be of great significance for immune function evaluation and immune intervention in sepsis.

Treg cells more likely not depend on glucose oxidation, but instead use exogenous fatty acids to promote oxidative phosphorylation through fatty acid oxidation.^11,15,21^ Treg cells have higher number of mitochondria with greater activity as compared to conventional CD4+T cells *ex vivo*.^22^ However, a study has found that, *ex vivo* Treg cells were highly glycolytic, whereas Treg cells engaged both glycolysis and fatty acid oxidation to proliferate *in vitro*.^23^ Some studies showed that Treg cells developing *in vivo* exhibit similar immune-metabolic phenotype with effector T (Teff) cells, they both use glycolysis-driven lipid synthesis to maintain cell proliferation and survival.^24,25^ Therefore, the metabolic characteristics of Treg cells *in vivo* and the metabolic reprogramming of Treg cells during severe infection may require further research.

In this paper, we used LC-MS/MS to analyse the metabolic profiles of freshly-isolated spleen Treg cells in sepsis mice and control mice. We found that the metabolic phenotype of sepsis Treg cells and the control group was different, and activated Treg cells have increased glycolysis and fatty acid oxidation (FAO). Inhibition of glycolysis and FAO have significant effects on the number and function of Treg cells, and intervene the metabolic process of Treg cells may improve the survival of sepsis mice. Our research suggests that the function of Treg cells undergoes significant changes during severe infections. These changes are accompanied by a series of metabolic reprogramming, and the metabolism may provide immune intervention targets for future sepsis treatment.

## 2. Materials and methods

### 2.1 Mice

Six to eight-week-old male C57BL/6 mice (20–25 g) were used for all experiments. All animals were cared for according to the Animal Care Guidelines of Capital Medical University Animal Committee. The study design was approved by Animal Department, Medical Research Center, Beijing Chaoyang Hospital, Capital Medical University (MRCCY-18070147-00, 21 07 2018).

### 2.2 Animal model of sepsis

The experimental animals were randomly divided into the experimental group (24 h, *n* = 36) and the sham operation group (*n* = 27). The animals were subjected to cecal ligation and puncture (CLP) as previously reported. Briefly, mice were anesthetized with a mixture of 5% chloral hydrate (80 mg kg^−1^) given intraperitoneally. Ligate cecum between distal pole and the basis of the cecum with a 3.0 suture, and perforated once with a 14-gauge needle. Extrude a small amount (droplet) of feces from both the mesenteric and antimesenteric penetration holes to ensure patency, relocate the cecum into the abdominal cavity. Close the peritoneum, fasciae and abdominal musculature by applying simple running sutures, close the skin by using metallic clips. The animals were resuscitated with normal saline (37 °C; 5 mL per 100 g body weight) after CLP. All animals were returned to their cages with free access to food and water. In the sham-operated group, the mice were submitted to all surgical procedures, but the cecum was neither ligated nor perforated, the mice were killed with neck fracture after 24 h for CLP or sham operation.

### 2.3 Splenic single-cell suspension preparation

Soaked the killed mice in 75% alcohol and removed the spleen. Use spleen dissociation kit (Miltenyi Biotec, mouse) to broke down spleen. Transfer one mouse spleen into the gentle MACS C tube containing A enzyme and D enzyme mixture. Tightly closed C tube and attached it onto the gentle MACS dissociator. Run the gentle MACS program m_spleen_02 about 8 s. Incubated sample for 15 min at 37 °C then run m_spleen_03 program. Centrifuged cell suspension at 300 g for 10 min, aspirate supernatant completely. Resuspend cells with buffer S, constant volume.

### 2.4 Magnetic activated CD4+CD25+ regulatory T cell sorting

The sorting process of CD4+CD25+ regulatory T cell includes the following four steps. First, magnetic labeling of non-CD4+ cells and fluorescent labeling of CD25+ cells. Second, depletion of non-CD4+ T cells. Third, magnetic labeling of CD25+ cells. And last, positive selection of CD4+CD25+ regulatory T cells.

### 2.5 Cell activity and purity verification

Flow cytometry was used to verify the cell activity and purity after magnetic beads separation. The separated cells were resuspended with DMSO solution and divided into isotype pair tube, CD4+CD25+ tube and sample tube. Added 1 μL FVS510 (BD biosciences, 564406) in three tubes. Added 2 μL PE-labelled CD4 Isotype (BD biosciences, clone A95-1, rat. # 553989) and 5 μL APC-labelled CD25 isotype (BD biosciences, clone A110-1, rat. # 550884) in isotype pair tube. Added 2 μL PE-labelled CD4 (BD biosciences, clone GK1.5, rat. # 561829) and 5 μL APC-labelled CD25 (BD biosciences, clone PC61, rat. # 561048) in CD4+CD25+ tube and sample tube. 1 mL of freshly prepared 1× TF Fix/Perm working buffer was added to each tube, which was mixed and incubated at 2–8 °C in dark for 30 min. Added 1 mL 1× Perm/Wash working buffer into each tube. Added 2 μL BV421-labeled Foxp3 antibody (BD biosciences, clone MF23, rat. # 562996) and 2 μL BV421-labeled Foxp3 isotype antibody (BD biosciences, clone R35-38, rat. # 562603) in sample tube and isotype pair tube, respectively. The solution was stained for 30 min avoiding light, and then was centrifugated for 5 min. Added 0.5 mL PBS into each tube for mixing.

### 2.6 Sample preparation

20 μg protein lysate per sample were reduced with 25 mM DTT at 60 °C for 30 min and alkylated with 50 mM iodoacetamide in the dark for 10 min. After alkylation, the sample was loaded on an ultrafiltration filter (10 kDa cutoff, Sartorius, German) for FASP digestion. Trypsin was added at a ratio of 1 : 100 (enzyme to protein) at 37 °C for 14–16 h. The samples were spun at 20 000 g at 4 °C for 10 min. The peptides were desalted using Ziptip C18 pipette tips (Merck KGaA, Darmstadt, Germany) according to manufacturer's instructions. After drying, the peptides were resuspended in 0.1% formic acid.

### 2.7 Mass spectrometric acquisition

1 μg of the samples was analyzed using a self-made analytical column (75 μm × 150 mm, 1.9 μm) on an EASY-nLC1000 connected to an Orbitrap Fusion Lumos mass spectrometer (Thermo Scientific). Peptides were eluted by using a binary solvent system with 99.9% H_2_O, 0.1% formic acid (phase A) and 80% ACN, 19.9% H_2_O, 0.1% formic acid (phase B). The following linear gradient was used: 2–5% B in 2 min, 5–10% B in 29 min, 10–20% B in 78 min, 20–28% B in 6 min, 28–95% B in 2 min, washed at 95% B for 3 min. The eluent was introduced directly to a mass spectrometer *via* EASY-Spray ion source. Source ionization parameters were as follows: spray voltage, 2.2 kV; capillary temperature, 320 °C; and decluttering potential, 100 V.

### 2.8 Mass spectrometric data analysis

The MS data were analyzed using MaxQuant software. MS data were searched against the Uniprot Mus database (54 448 entries). An initial search was set at a precursor ion mass tolerance of 6 ppm for peptide masses and a mass tolerance of 20 ppm for fragment ions. The search was performed with enzyme specificity trypsin, and two missed cleavages were allowed. Carbamidomethylation of cysteines was defined as fixed modification, while protein N-terminal acetylation and methionine oxidation were defined as variable modifications for database searching. The cutoffs of global false discovery rate (FDR) for both peptide and protein identification were set to 0.01. The protein identification based on N1 unique peptides, and minimal peptide length was six amino acids. The protein abundance was calculated using intensity-based absolute quantification (iBAQ) in MaxQuant software.

### 2.9 2-Deoxy-d-glucose (2-DG)/etomoxir treatment

Mice were treated with 0.5 g kg^−1^ 2-DG three hours prior to CLP.^26^ Etomoxir (Sigma, St.) was dissolved in sterile water. After sepsis induction, mice received intraperitoneal injections of 15 mg kg^−1^ (ref. 27) in a volume of 100 μL every day and last for four days. Control (vehicle-treated) mice received sterile water only.

### 2.10 Western blot analysis

In brief, after protein extraction, proteins in cell lysates were first resolved by SDS-polyacrylamide gel electrophoresis and then transferred to polyvinylidene difluoride membrane and subsequently incubated with the primary antibody. The antibodies were used the following: anti-PKM1/2 (1 : 1000 dilution, Proteintech), anti-HADHA (1 : 2500 dilution, Proteintech), anti-FoxP3 (1 : 1000 dilution, Proteintech), anti-GAPDH (1 : 20 000 dilution, Proteintech). After incubation with peroxidase-conjugated secondary antibodies (1 : 2000), the signals were visualized by enhanced chemiluminescence according to the manufacturer's instructions.

### 2.11 Apoptosis detection

Washed cells twice with cold cell staining buffer, and then resuspend cells in Annexin V binding buffer at a concentration of 1 × 10^6^ cell per mL. Transferred 100 μL of cell suspension in a 5 mL test tube (unstained group, single-stained Annexin V group, single-stained PI group, PI and Annexin V double-stained control group, and sample group). Added 5 μL of APC Annexin V. Added 5 μL of 7-AAD Viability Staining Solution. Gently vortexed the cells and incubated for 15 min at room temperature (25 °C) in the dark. Added 400 μL of Annexin V binding buffer to each tube.

### 2.12 Statistical analysis

The Perseus software was used to analyze the differences of proteins in proteomic inventories between CLP (24 h) and sham (24 h). The data were subjected to an analysis of variance (two-sample *t*-tests). It was set 1.3-fold for up-changed and 0.77-fold for down-changed cut off values. Data were analyzed by a 2-tailed Student's *t* test between two samples. The results were expressed as mean ± SEM and the differential changes were considered significant at *P* < 0.05.

## 3. Results and discussion

### 3.1 Freshly-isolated mice spleen Treg cells have high activity and purity

Immune disorders during sepsis directly affect the patient's prognosis.^2^ It is generally believed that immunosuppression in the later stages of sepsis is related to the loss of effector T cells and the upregulation of Treg cells. Treg cells play a major role in suppressing early hyperinflammatory responses and late immunosuppression in sepsis. The fate and function of Treg cells are affected by their metabolic status,^19,20^ but most of the current research on Treg metabolism is induced *in vitro*, which cannot reflect the true metabolic status of the body. Therefore, it is necessary to study the metabolic characteristics of Treg cells *in vivo*.

A stable and successful model of sepsis is the basis of research. The cecal ligation puncture (CLP) method used in this study is the internationally recognized gold standard for animal models of sepsis. In this experiment, a moderate ligature was used, and a No. 4 needle for puncture (Fig. S1a and S1b†). The 72 h survival rate in sepsis mice was about 50%, and in the sham operation group was 100% (Fig. 4f), which is consistent with that reported in the literature.^28^ The model was established after 24 h, and the levels of inflammatory factors IL-6, TNF-α, and IL-10 in peripheral blood of mice were detected. Compared with the sham operation group, the pro-inflammatory and anti-inflammatory factors in CLP mice were significantly increased (Fig. S1c†), suggesting the existence of a disordered immune function in sepsis group.

**Fig. 1:**
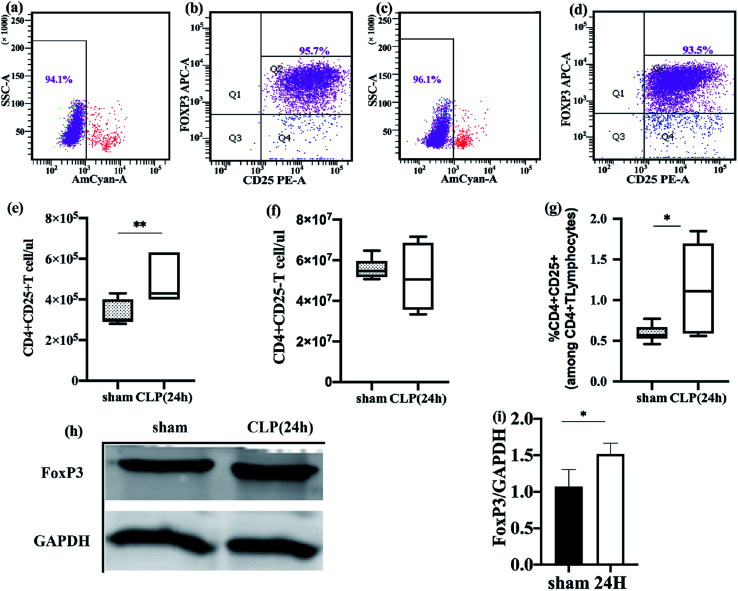
Freshly-isolated splenic Treg cells in sepsis mice are increased. (a–d) Freshly-isolated cells were labeled with CD4+, CD25+, and Foxp3 antibodies, then, the viability and purity of Treg cells in sepsis group (a and b) and sham group (c and d) were detected by flow cytometry. (e and f) Absolute counting of regulatory T (CD4+25+FoxP3+, Treg) lymphocytes (e) and effector T (CD4+CD25−, Teff) lymphocytes (f) were performed in spleen from sepsis mice (open box, *n* = 36) and sham mice (gray box, *n* = 27). (g) CD4+T cells were examined for their percentage of Treg cells, sepsis group was represented by open box and gray box represented sham group. (h and i) FoxP3 expression in sepsis group and sham group. The data are shown as mean ± SEM of 3 independent experiments, each of them in duplicates. (*p < 0.05, **p < 0.01).

To more accurately reflect the metabolic status of Treg cells *in vivo*, we need to guarantee the activity and purity of freshly isolated Treg cells. After sorting, multiple verifications were performed using flow cytometry, and it was found that the purity of CD4+ CD25+ Foxp3+ Treg cells in the control group and experimental group after two MACS sorting can reach 92.13–95.6% (Fig. 1b and d), CD4+ CD25+ Treg cells stained with Fixable Viability Stain 510 and detected cell viability exceeding 94% (Fig. 1a and c). By strictly controlling the operation and verification, the freshly isolated Treg can basically represent its state in the body. Freshly isolated Treg cells were immediately frozen with liquid nitrogen, followed by mass spectrometry.

### 3.2 The percentage of freshly-isolated splenic Treg cells was significant increase in sepsis mice

Immune dysfunction plays an important role in the occurrence and development of sepsis, and Treg cells have an important regulatory role in this immune disorder.^29^ To better understand the changes of Treg cells during sepsis, we counted freshly isolated Treg cells and effector T cells. The function of Treg cells was also studied.

Compared to the sham-operated mice, the absolute counts and percentages of Treg in the spleen of sepsis mice increased (Fig. 1e and g). The effector T cell count was not significantly different between the two groups (Fig. 1f). The fork-head/winged helix transcription factor p3 (FoxP3) specifically expressed in Treg cells was also significantly different between the two groups, and the expression level of FoxP3 in the sepsis group was increased (Fig. 1h and i). This is consistent with the results of previous studies.^30^ Flow cytometry was used to detect the apoptosis of freshly isolated splenic Treg cells. It was found that the rate of apoptosis of spleen Treg cells in sepsis mice was significantly lower than that in the sham operation group (Fig. 4c). This finding suggests that Treg cells are more resistant to sepsis-induced apoptosis. This increase was observed immediately after the onset of sepsis but persisted only in those patients who subsequently died.^31^

Tregs are associated with sepsis-associated immune-paralysis.^32^ After the onset of septic shock, the number of Tregs and their suppressive function were increased progressively and significantly.^31,33^ Subsequent research suggested that this relative increase was due to a decrease in effector T cell numbers rather than changes in the absolute numbers of Treg cells.^34^ In our study, we mainly observed the count and the percentage of Treg cells in the spleen of sepsis mice. Like previous research results, the percentage of spleen Treg cells and FoxP3 expression are increased during sepsis, and the apoptosis rate was significantly lower than that of non-septic group. We also counted the absolute counts of Treg cells and effector T cells, and in the sepsis group the absolute count of Treg cells showed increased, which is inconsistent with the results of the study by Venet *et al.*^34^ The reasons for this difference may have the following aspects. First, the research by Venet *et al.* was based on Treg cells in human peripheral blood, and we focused on the CD4+ CD25+ T cell subpopulation in the mouse spleen. The distribution of subpopulations of cells in the spleen is different from that in peripheral blood. In the study of Scumpia *et al.*,^35^ the number of Treg cells in the spleen of sepsis mice also increased significantly, which is consistent with our results. Secondly, the distribution of T lymphocytes may be related to species differences. Finally, this may be caused by insufficient sample size.

### 3.3 Splenic Treg cells in sepsis and non-septic groups showed different protein profiles

Based on the important role of Treg cells in the development of sepsis, we examined the metabolic protein profiles of Treg cells in the control and experimental groups in detail to compare whether there are differences in metabolic related proteins between the two. The protein expression profiles of two freshly isolated Treg cell populations were measured by high resolution LC-MS/MS. A total of 2697 proteins were identified, of which 241 were differential proteins. The identified proteins and differentially expressed proteins are listed in Table S1 and S2.† The most representative functional classes that we had found differentially expressed between two groups of freshly-isolated Treg cells were those associated with metabolism.

**Fig. 2:**
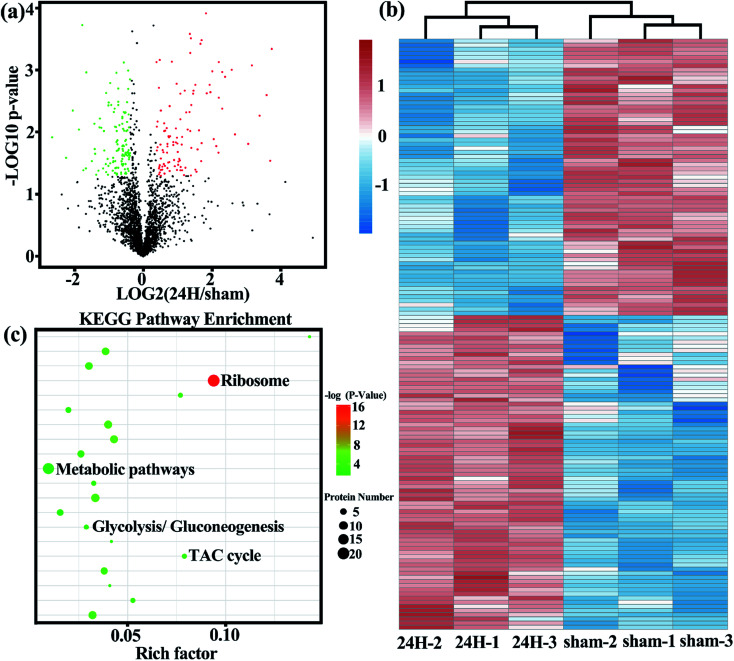
The Treg cells in the sepsis group and the non-sepsis group showed different protein profiles. Sorted cell's lysates from 3 biological replicate samples were extracted and analyzed using high-resolution LC-MS/MS for determination of cellular proteins. (a) Volcano plot show the difference of protein expression levels between the two groups. Each point represents a protein. Red dots correspond to specific proteins upregulated in sepsis (24 h) Treg cells; green dots correspond to proteins down-regulated in sepsis (24 h) Treg cells; grey dots represent non-differentially expressed proteins between the two group. (b) Showing the relative levels of metabolism-related differential protein and unsupervised hierarchical clustering from independent biological replications. (c) KEGG pathway enrichment (bubble chart). Bubble diagram was made for the first 20 KEGG pathways enriched (in the order of *P* value from small to large). The vertical axis represents the pathway name, and the horizontal axis represents the rich factor (the ratio of the number of differential proteins enriched in the pathway to the number of annotation proteins). The higher the enrichment factor, the more significant the enrichment level of differentially expressed proteins in this pathway. Each dot represents a KEGG pathway, the size of the dot represents the number of proteins enriched in the pathway, and the color of the dot represents *P* value. The smaller the *P* value, the more reliable the enrichment significance of differentially expressed proteins in the pathway.

The volcano plot (Fig. 2a) more intuitively shows the differences in protein expression levels between the two groups, and the statistical significance of the differences. The larger the absolute value of the abscissa, the greater the difference in protein expression between the two groups of samples; the larger the value of the ordinate, the more significant the differential expression, and the more reliable the differentially expressed protein obtained by screening. Unsupervised cluster analysis of 167 metabolic-related differential proteins showed that the spleen Treg cells in the sepsis group and non-septic group had extensive metabolic differences (Fig. 2b). We analyzed the gene list for enrichment of gene ontology and canonical pathways using the Cluster Profiler bioinformatics databases. The first few pathways with the most significant enrichment were related metabolic pathways (Fig. 2c).

### 3.4 Comparison of metabolic related differential proteins between sepsis and non-septic groups

Through detailed proteomic analysis, we found that the spleen Treg cells in sepsis mice had extensive metabolic differences compared with non-septic mice. Compared with non-septic group, spleen Treg cells in septic mice are upregulated (Fig. 2b, Table S2†), such as ATP-dependent 6-phosphofructokinase [ATP-PFK], aldolase A [ALDOA], enolase 1 [ENO1], hexokinase [HK]. The increase is agreement with the high proliferative profile of these cells *in vivo*.^23,36^

**Fig. 3:**
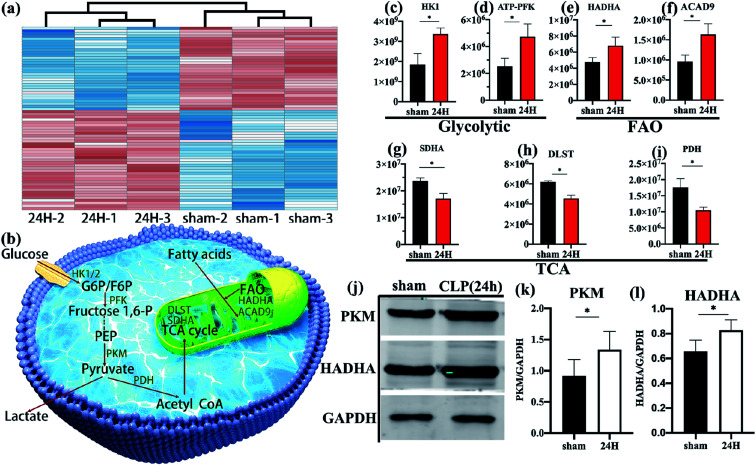
Comparison of different metabolism-related proteins between sepsis group and non-sepsis group. (a) The mitochondrial related proteins were analyzed by hierarchical clustering. (b) Schematic diagram of the relationship between several metabolic pathways. Quantitative differential protein, (c and d) Comparison of glycolysis-related key enzymes in Treg cells in sepsis group and non-septic group. (e and f) Comparison of key fatty acid oxidation enzymes in the two groups. (g–i) Comparison of pyruvate dehydrogenase and key tricarboxylic acid cycle enzymes in the two groups. (j–l) Immunoblot for PKM1/2 and HADHA normalized on GAPDH in freshly-isolated Treg cells in sepsis group and non-septic group. Data are shown as mean ± SEM of triplicate samples, and all data are representative of at least three independent experiments. *P < 0.05.

Unsupervised cluster analysis of mitochondrial genes showed that compared with the non-septic group, the sepsis Treg expressed a higher level of mitochondrial metabolic protein (Fig. 3a). An upregulation of fatty acid oxidation-related proteins: enoyl-CoA delta isomerase 1 [ECI1], catalytic trifunctional enzyme [ECHA], hydroxyacyl-CoA dehydrogenase [HADHA], and acyl-CoA dehydrogenase [ACAD9]. Downregulation of enzymes involved in the Krebs tricarboxylic acid cycle (TCA): succinate dehydrogenase [SDHA], proteins involved in the mitochondrial respiratory electron transport chain (electron transfer flavoprotein [ETFA]), succinate-CoA ligase [SUCB1] involved in ATP synthesis, NADH dehydrogenase family on mitochondrial membrane involved in electronic respiratory chain transfer ([NDUS2] [E9Q9] [NDUV2]). It is worth noting that higher levels of pyruvate dehydrogenase [PDH] are present in non-septic Treg cells, indicating that they preferentially oxidize pyruvate.

In addition, the lipid metabolism-related proteins in Treg of sepsis mice were higher than those in the control group, including APOA1, serpin peptidase inhibitor member 3 (Serpin A3). Consistent with results from proteomics studies of fresh Treg cells in humans.^23^

### 3.5 Freshly-isolated splenic Treg cells in sepsis mice showed high glycolysis and fatty acid oxidation

Schematic diagram (Fig. 3b) showing several major metabolic pathways and their key regulatory enzymes. According to the quantitative results of differential proteins, the key regulatory proteins of glycolysis (hexokinase [HK], 6-phosphofructokinase [ATP-PFK], Fig. 3c and d) and fatty acid oxidation (HADHA, ACAD9, Fig. 3e and f) of spleen Treg cells in sepsis mice were significantly higher than those in the control group. The key enzymes of the tricarboxylic acid cycle (SDHA, dihydrolipoamide succinyl-transferase [DLST], Fig. 3g and h), and the pyruvate dehydrogenase (Fig. 3i) in the sepsis group were significantly reduce. This indicates that the metabolic pathways of activated Treg cells have undergone reorganization, from aerobic oxidation to glycolysis and fatty acid oxidation.

These data were confirmed at the biochemical level. The glycolytic key enzyme pyruvate kinase (PKM) and the fatty acid oxidation key enzyme HADHA were increased in the sepsis group (Fig. 3j–l).

*In vitro* conditions, naturally occurring Tregs have unique metabolic characteristics,^37^ and the instability of the Treg cell lineage is closely linked to alterations in metabolism.^38,39^ Studies have found that the expression level of GLUT1 on the surface of Tregs is lower than that of Teff cells.^37^ Tregs have higher number of mitochondria with greater function/activity.^22^ Therefore, it is believed that Tregs are not dependent on glycolytic metabolism. In contrast, these natural Tregs exhibit high lipid oxidation rates under *in vitro* conditions.^11^ Blocking glycolysis inhibits proinflammatory TH17 cell development but promotes the generation of Treg cells.^15^ Interestingly, a proteome-based study found that isolated Tregs are highly glycolytic and that proliferation *in vitro* depends on glycolysis and fatty acid oxidation.^23^ Treg cells that develop *in vivo* resemble Teff cells in that they depend on glycolysis-driven lipogenesis for their proliferation and functional fitness.^25^ A study of mouse B16 melanoma model found that Treg cells in tumors and spleen showed higher glucose uptake compared to effector T cells.^40^ Our research also found that during sepsis, the glycolysis level of Treg cells in the mice spleen is increased. This increase may be related to the proliferation and effector function of Treg cells upon activation. In this study, we also found that fatty acid oxidation-related proteins were also significantly upregulated in spleen Treg cells of sepsis mice, which is like the results of some *in vitro* studies.^11,19^ These studies indicate that there may be significant differences in the metabolic phenotypes of naturally occurring Tregs *in vivo* and *in vitro*, which are related to the environment, physiological/pathological state of Treg cells, different species, and different anatomical locations.

### 3.6 Inhibition of glycolysis and fatty acid oxidation alters the number and function of mice Treg cells

**Fig. 4:**
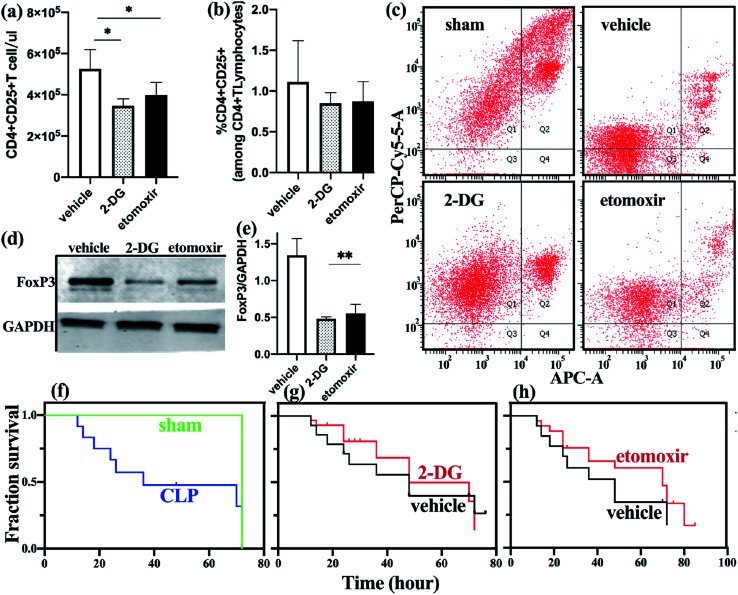
Blocking glycolysis and fatty acid oxidation increases Treg apoptosis and improves early survival outcome. Inhibitor group mice were treated with 0.5g kg^−1^ 2-DG three hours prior to CLP or treated with etomoxir (15 mg kg^−1^) every day after induction of sepsis. Vehicle-treated mice received sterile water only. (a) Absolute counting of Treg cells in etomoxir treatment group and control group. (b) The percentage of Treg cells on the CD4+T cells in different group. (c) The degree of apoptosis of freshly-isolated Treg cells in the three groups were assessed by flow cytometry. (d and e) Immunoblot for FoxP3 normalized on GAPDH in freshly-isolated Treg cells in vehicle-treated group and inhibitor group. (f) Survival analysis of sepsis group (*n* = 36) and non-septic group (*n* = 27). (g) Comparison of survival rate between vehicle group and 2-DG group (*n* = 19). (h) Comparison of survival rate between vehicle group and etomoxir group (*n* = 24).

To determine the effect of metabolic disorders on mouse spleen Treg cells, we treated sepsis mice with either 2-DG or etomoxir and observed changes in the number and function of freshly isolated splenic Treg cells. Compared with the untreated group, the absolute count of Treg cells in the spleen of sepsis mice treated with 2-DG or etomoxir was decreased (Fig. 4a), but the percentage of Treg cells was not significantly different from the untreated group (Fig. 4b). Inhibition of glycolysis and fatty acid oxidation, absolute count of Treg cells in spleen of sepsis mice is significantly reduced. However, the percentage of Treg cells have no significantly different among the three groups, probably because the number of effector T cell subsets also decreased after inhibiting metabolism. T cell subsets require further analysis by flow cytometry.

To further determine the changes of Treg cells in the spleen of sepsis mice, we studied the apoptotic function of freshly isolated Treg cells and found that the apoptosis of spleen Treg cells in the sepsis group was significantly lower than the non-septic group. Sepsis mice with 2-DG or etomoxir treatment showed an increase in the apoptosis level of Treg cells, especially in the 2-DG treatment group (Fig. 4c), indicating that inhibition of glycolysis or fatty acid oxidation of Treg cells can increase their apoptosis levels.

To better understand the functional changes of Treg cells, we studied the effect of inhibitors on the expression of FoxP3 in Treg cells. FoxP3 expression was reduced in Treg cells treated with 2-DG and etomoxir, as compared to c untreated group (Fig. 4d and e). This result is consistent with the *in vitro* studies of Claudio *et al.*^23^

We observed the effect of inhibitors on the survival rate of sepsis mice as shown in Fig. 4g and h. Although there was no statistically significant difference in survival rates between the treated and non-treated mice, we found that non-treated mice began to die at 15 h after CLP, and 50% of the mice died after 48 h of CLP. Etomoxir-treated sepsis mice started to die at 17 h after CLP, but their median survival time (time to 50% mortality) was extended to 70 h. In addition, the survival rates of the sepsis mice in the treatment group at 48 h and 72 h were 65% and 45%, respectively, which were higher than those in the non-treatment group. 2DG-treated group and no-treated group have no significant difference in survival analysis. These results indicate that etomoxir may have beneficial effects on the survival outcome of CLP-induced sepsis mice, but the specific impact results need further large-scale research.

All the results showed that specific inhibition of glycolysis and FAO metabolism can inhibit the proliferation and effector function of Treg cells in the body. The two metabolic programs are essential for Treg cells in sepsis mice.

## 4. Conclusions

In conclusion, our study revealed the true metabolic status of splenic Treg cells in the later stages of sepsis, Treg cell activation may depend on glycolysis and fatty acid oxidation. This metabolic change is critical to the number and function of Treg cells, and intervention in Treg cell metabolism may improve the survival time of mice. These results are meaningful for grasping the function and overall immune status of Treg during sepsis, and also provide new ideas for the future immunotherapy of sepsis.

## Abbreviations

2-DG: 2-Deoxy-d-glucose
ACAD9: Acyl-CoA dehydrogenase
ALDOA: Aldolase A
APOA1: Apolipoprotein1
ATP-PFK: ATP-dependent 6-phosphofructokinase
CLP: Cecal ligation and puncture
ECHA: Trifunctional enzyme
ECI1: Enoyl-CoA delta isomerase 1
ENO1: Enolase 1
ETFA: Electron transfer flavoprotein
FAO: Fatty acid oxidation
FoxP3: Fork head/winged helix transcription factor p3
GAPDH: Glyceraldehyde-3-phosphate dehydrogenase
GLUT1: Glucose transporter type 1
HADHA: Hydroxyacyl-coenzyme A dehydrogenase
HK: Hexokinase
IL-10: Interleukin-10
IL-6: Interleukin-6
kDa: Kilodalton
LC/MS: Liquid chromatography/mass spectrometry
PDH: Pyruvate dehydrogenase
PI: Propidine iodide
PKM: Pyruvate kinase
SDHA: Succinate dehydrogenase
Serpin A3: Serpin peptidase inhibitor member 3
SUCB1: Succinate-CoA ligase
TCA cycle: Tricarboxylic acid cycle
Teff: Effector T Cell
TNF-α: Tumor necrosis factor-α
Treg: Regulatory cells

## Conflicts of interest

The authors declare no conflict of interest.

## Supplementary Material

RA-010-D0RA01947J-s001

RA-010-D0RA01947J-s002

RA-010-D0RA01947J-s003

RA-010-D0RA01947J-s004

RA-010-D0RA01947J-s005

## References

[cit1] Armstrong B. A., Betzold R. D., May A. K. (2017). Surg. Clin. North Am..

[cit2] Hotchkiss R. S., Monneret G., Payen D. (2013). Nat. Rev. Immunol..

[cit3] Munford R. S., Pugin J. M. (2001). Am. J. Respir. Crit. Care Med..

[cit4] Hotchkiss R. S., Coopersmith C. M., McDunn J. E., Ferguson T. A. (2009). Nat. Med..

[cit5] Otto G. P., Sossdorf M., Claus R. A., Rodel J., Menge K., Reinhart K., Bauer M., Riedemann N. C. (2011). Critical Care.

[cit6] Ward P. A. (2012). EMBO Mol. Med..

[cit7] Boomer J. S., To K., Chang K. C., Takasu O., Osborne D. F., Walton A. H., Bricker T. L., Jarman 2nd S. D., Kreisel D., Krupnick A. S., Srivastava A., Swanson P. E., Green J. M., Hotchkiss R. S. (2011). JAMA.

[cit8] Weber G. F., Swirski F. K. (2014). Langenbeck's Arch. Surg..

[cit9] Tatura R., Zeschnigk M., Hansen W., Steinmann J., Vidigal P. G., Hutzler M., Pastille E., Westendorf A. M., Buer J., Kehrmann J. (2015). Immunology.

[cit10] Kumar V. (2018). Eur. J. Cell Biol..

[cit11] Michalek R. D., Gerriets V. A., Jacobs S. R., Macintyre A. N., MacIver N. J., Mason E. F., Sullivan S. A., Nichols A. G., Rathmell J. C. (2011). J. Immunol..

[cit12] Macintyre A. N., Gerriets V. A., Nichols A. G., Michalek R. D., Rudolph M. C., Deoliveira D., Anderson S. M., Abel E. D., Chen B. J., Hale L. P., Rathmell J. C. (2014). Cell Metab..

[cit13] Donnelly R. P., Loftus R. M., Keating S. E., Liou K. T., Biron C. A., Gardiner C. M., Finlay D. K. (2014). J. Immunol..

[cit14] Doughty C. A., Bleiman B. F., Wagner D. J., Dufort F. J., Mataraza J. M., Roberts M. F., Chiles T. C. (2006). Blood.

[cit15] Shi L. Z., Wang R., Huang G., Vogel P., Neale G., Green D. R., Chi H. (2011). J. Exp. Med..

[cit16] Clever D., Roychoudhuri R., Constantinides M. G., Askenase M. H., Sukumar M., Klebanoff C. A., Eil R. L., Hickman H. D., Yu Z., Pan J. H., Palmer D. C., Phan A. T., Goulding J., Gattinoni L., Goldrath A. W., Belkaid Y., Restifo N. P. (2016). Cell.

[cit17] Sun L., Fu J., Zhou Y. (2017). Front. Immunol..

[cit18] Weyand C.
M., Goronzy J. J. (2017). Nat. Rev. Rheumatol..

[cit19] Gerriets V. A., Kishton R. J., Nichols A. G., Macintyre A. N., Inoue M., Ilkayeva O., Winter P. S., Liu X., Priyadharshini B., Slawinska M. E., Haeberli L., Huck C., Turka L. A., Wood K. C., Hale L. P., Smith P. A., Schneider M. A., MacIver N. J., Locasale J. W., Newgard C. B., Shinohara M. L., Rathmell J. C. (2015). J. Clin. Invest..

[cit20] O'Neill L. A., Kishton R. J., Rathmell J. (2016). Nat. Rev. Immunol..

[cit21] Wang H., Flach H., Onizawa M., Wei L., McManus M. T., Weiss A. (2014). Nat. Immunol..

[cit22] Beier U. H., Angelin A., Akimova T., Wang L., Liu Y., Xiao H., Koike M. A., Hancock S. A., Bhatti T. R., Han R., Jiao J., Veasey S. C., Sims C. A., Baur J. A., Wallace D. C., Hancock W. W. (2015). FASEB J..

[cit23] Procaccini C., Carbone F., Di Silvestre D., Brambilla F., De Rosa V., Galgani M., Faicchia D., Marone G., Tramontano D., Corona M., Alviggi C., Porcellini A., La Cava A., Mauri P., Matarese G. (2016). Immunity.

[cit24] Newton R., Priyadharshini B., Turka L. A. (2016). Nat. Immunol..

[cit25] Zeng H., Yang K., Cloer C., Neale G., Vogel P., Chi H. (2013). Nature.

[cit26] Zheng Z., Ma H., Zhang X., Tu F., Wang X., Ha T., Fan M., Liu L., Xu J., Yu K., Wang R., Kalbfleisch J., Kao R., Williams D., Li C. (2017). J. Infect. Dis..

[cit27] Raud B., Roy D. G., Divakaruni A. S., Tarasenko T. N., Franke R., Ma E. H., Samborska B., Hsieh W. Y., Wong A. H., Stuve P., Arnold-Schrauf C., Guderian M., Lochner M., Rampertaap S., Romito K., Monsale J., Bronstrup M., Bensinger S. J., Murphy A. N., McGuire P. J., Jones R. G., Sparwasser T., Berod L. (2018). Cell Metab..

[cit28] Rittirsch D., Huber-Lang M. S., Flierl M. A., Ward P. A. (2009). Nat. Protoc..

[cit29] Kessel A., Bamberger E., Masalha M., Toubi E. (2009). J. Autoimmun..

[cit30] Bao R., Hou J., Li Y., Bian J., Yang T. (2016). Am. J. Transl. Res..

[cit31] Monneret G., Debard A. L., Venet F., Bohe J., Hequet O., Bienvenu J., Lepape A. (2003). Crit. Care Med..

[cit32] Cao C., Ma T., Chai Y. F., Shou S. T. (2015). World J. Emerg. Med..

[cit33] Taylor A. L., Llewelyn M. J. (2010). J. Immunol..

[cit34] Venet F., Pachot A., Debard A. L., Bohe J., Bienvenu J., Lepape A., Monneret G. (2004). Crit. Care Med..

[cit35] Scumpia P. O., Delano M. J., Kelly K. M., O'Malley K. A., Efron P. A., McAuliffe P. F., Brusko T., Ungaro R., Barker T., Wynn J. L., Atkinson M. A., Reeves W. H., Salzler M. J., Moldawer L. L. (2006). J. Immunol..

[cit36] Vukmanovic-Stejic M., Zhang Y., Cook J. E., Fletcher J. M., McQuaid A., Masters J. E., Rustin M. H., Taams L. S., Beverley P. C., Macallan D. C., Akbar A. N. (2006). J. Clin. Invest..

[cit37] Zeng H., Chi H. (2015). Trends Immunol..

[cit38] Huynh A., DuPage M., Priyadharshini B., Sage P. T., Quiros J., Borges C. M., Townamchai N., Gerriets V. A., Rathmell J. C., Sharpe A. H., Bluestone J. A., Turka L. A. (2015). Nat. Immunol..

[cit39] Delgoffe G. M., Woo S. R., Turnis M. E., Gravano D. M., Guy C., Overacre A. E., Bettini M. L., Vogel P., Finkelstein D., Bonnevier J. (2013). Nature.

[cit40] Ho P. C., Bihuniak J. D., Macintyre A. N., Staron M., Liu X., Amezquita R., Tsui Y. C., Cui G., Micevic G., Perales J. C., Kleinstein S. H., Abel E. D., Insogna K. L., Feske S., Locasale J. W., Bosenberg M. W., Rathmell J. C., Kaech S. M. (2015). Cell.

