# Supplementary material for: Glycolytic and lipid oxidative metabolic programs are essential for freshly-isolated regulatory T cells in mice with sepsis
Source: RSC Adv. 2020 Jun 3;10(35):21000–8. doi: 10.1039/d0ra01947j (PMC9054272; doi:10.1039/d0ra01947j)
Supplement: RA-010-D0RA01947J-s003 [file RA-010-D0RA01947J-s003.pdf]

Electronic Supplementary Material (ESI) for RSC Advances.
This journal is © The Royal Society of Chemistry 2020

Glycolytic and Lipid Oxidative Metabolic Programs Are Essential for Freshly-Isolated Regulatory T Cell in Sepsis Mice

*Xiaomei Zhu, WenQing Ji, Shubin Guo, *, Di Zhu, Yue Yang, Xin Liu*

Beijing Key Laboratory of Cardiopulmonary-Cerebral Resuscitation, Emergency Department, Chao-Yang Hospital, Capital Medical University, Chaoyang District, Beijing 100020, China

** To whom correspondence should be addressed.*

Email: shubinguo@126.com

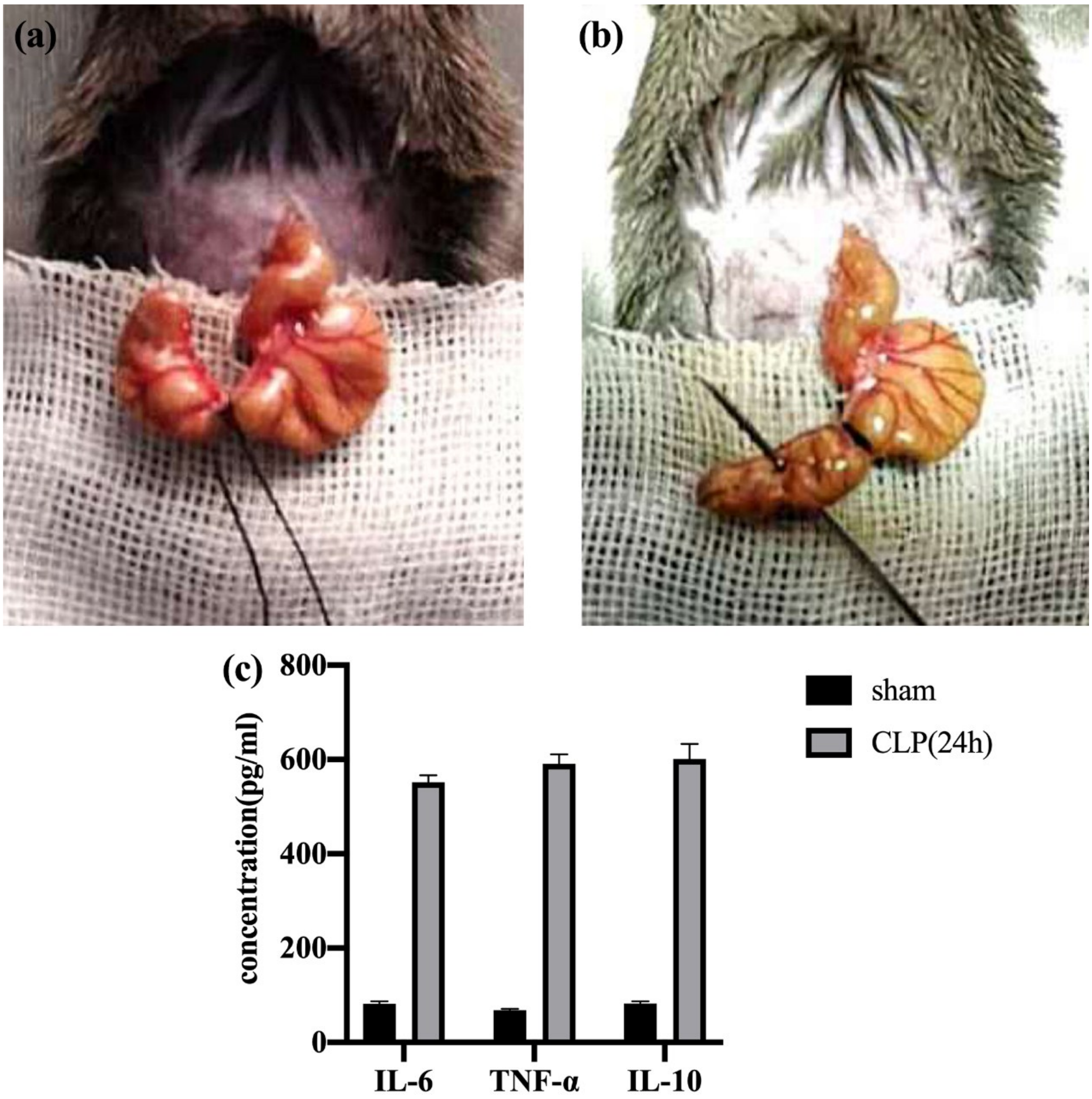

Fig. S1 (a) ·Ligation of the cecum at moderate position. (b) ·Cecal puncture ('through-and-through') use No. 4 needle. (c) Comparison of pro-inflammatory and anti-inflammatory factors between the two groups.

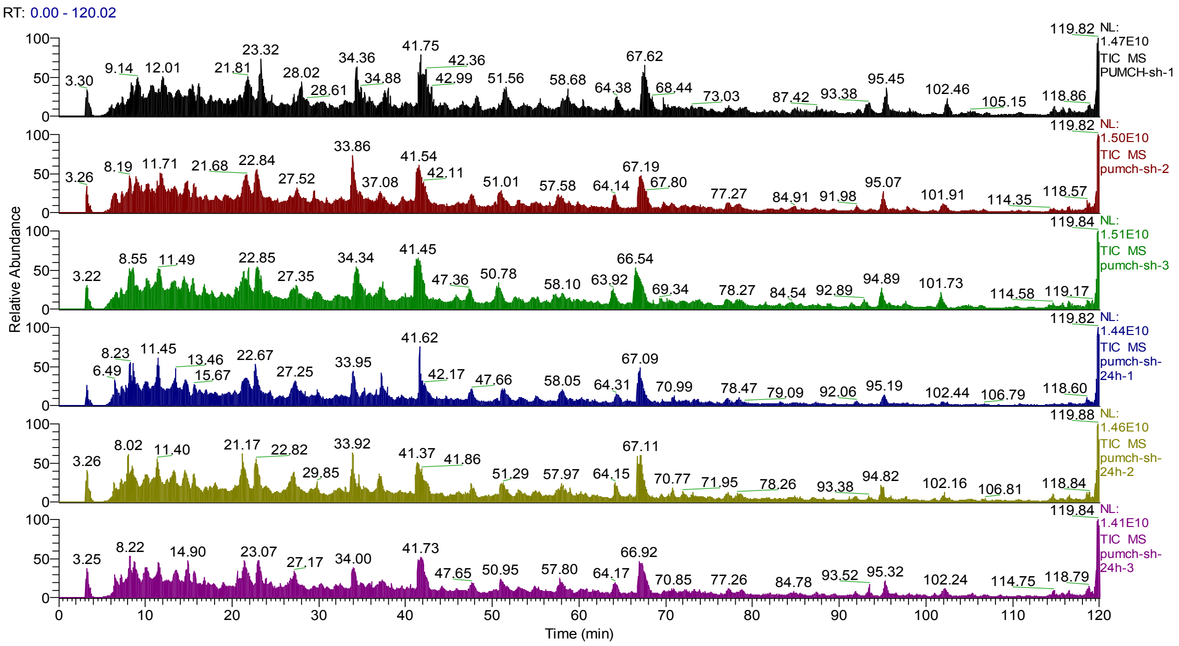

Fig. S2 The total ion chromatogram (TIC)

Three parallel samples were set in CLP group and sham group respectively. This figure shows the TIC of these 6 samples. We can see that the consistency of these 6 samples is very high, indicating that the mass spectrometry quantitative results are relatively accurate.
