# Supplementary material for: Glycolytic and lipid oxidative metabolic programs are essential for freshly-isolated regulatory T cells in mice with sepsis
Source: RSC Adv. 2020 Jun 3;10(35):21000–8. doi: 10.1039/d0ra01947j (PMC9054272; doi:10.1039/d0ra01947j)
Supplement: RA-010-D0RA01947J-s005 [file RA-010-D0RA01947J-s005.pdf]

Electronic Supplementary Material (ESI) for RSC Advances.
This journal is © The Royal Society of Chemistry 2020

iBAQ Intensity

T: Protein IDs	24H-1	24H-2	24H-3	sham-1	sham-2	sham-3	Student's T-test p-value sham_24H	Ratio(24H/sham)
sp|Q6ZWZ2	NaN	149095	148094	846959	1020750	NaN	0.0120252	0.1591195
sp|Q8BRF7|	216898	770279	110748	2161190	1067120	1946370	0.0257025	0.2121725
sp|Q9JJL8|S	113822	170414	NaN	507706	617552	643723	0.0044754	0.2410167
sp|Q9CR41|	1286420	3697510	1029540	8628930	6080290	7287510	0.0090435	0.2733802
sp|Q8K157|	4791560	3619160	3929260	15101400	14121800	12816300	0.0001894	0.293533
tr|A0A0A6Y	61455.9	197278	1289370	1552500	1938270	1665530	0.0412058	0.3002354
tr|A0A0R4J	247726	488737	361703	1091380	1624670	760761	0.0385334	0.3158544
sp|Q91XV3|	2288930	3112590	3793720	9455360	8687440	10915700	0.0010825	0.3164389
tr|E9PZ58|E	354567	560287	830210	1127910	1679960	2121110	0.0290775	0.3540416
sp|P26011|I	160221	267850	208430	481795	530731	691285	0.0072542	0.3735749
tr|K3W4R2|	82179.6	NaN	123184	221513	292998	303081	0.0187173	0.3767715
sp|Q9CQJ8|	3166010	3406660	2071330	8363280	5181280	9264540	0.0223882	0.3789716
sp|Q9D1M0|	1998150	2170320	8003490	10315100	8555050	12555000	0.0485697	0.3873318
tr|A0A3Q4L	4942040	6400980	5976600	11252300	11560600	19867800	0.0415093	0.4057951
tr|V9GX42|	5944370	6252910	5335230	10911900	14749700	16637500	0.0084042	0.414489
sp|O09000|	469468	259601	527244	1134930	981279	883855	0.0060198	0.4187621
sp|Q8C0E2|	778471	742885	1541900	2170600	2489990	2459150	0.0084406	0.4302483
tr|G5E8V8|	437663	325882	381129	1153240	739506	667147	0.0381386	0.447157
sp|O35638|S	164251	341932	285361	660898	580259	508014	0.0096042	0.4525252
sp|E9QAT4|	451204	590304	160600	896158	852469	893405	0.0196348	0.4549937
sp|Q91V41|	1173070	1308360	1306790	3408560	1881600	2783650	0.0326888	0.4691986
sp|Q62018|	257618	60527.2	212140	371676	318979	428358	0.0436726	0.4738865
tr|A0A075B	20413000	25739200	25565900	44327400	56151500	50540000	0.0023245	0.4748949
sp|O35730|R	214537	377506	400581	608442	847022	583870	0.0270356	0.4867393
sp|Q8VBT9|	363319	428715	219166	841347	740937	479352	0.048078	0.4904843
sp|Q9Z0H8|	37650.8	NaN	47887.5	100003	70096.6	89744.6	0.0350395	0.4937861
tr|F8WHU9|	1123110	1684690	1304210	3049580	2751540	2499770	0.0036848	0.4953698
tr|F7ABX5|	1376560	1260960	1410820	2971000	2870300	2289030	0.0033195	0.4979306
sp|Q8VD62|	1552270	2074030	1279660	3246340	3874590	2430710	0.0317975	0.5136249
sp|Q9DBG5|	3041450	2229460	2622520	5544910	4790570	4981590	0.0016145	0.5153355
sp|Q91WD5	3932240	2903080	3465500	6098470	7390450	6187760	0.0036428	0.523504
sp|Q9CX99|	NaN	751617	659583	1567150	1126760	1226220	0.0417069	0.5399821
sp|Q6PGL7|	442927	381843	286672	783249	729499	529244	0.0258322	0.544293
sp|Q67FY2|	160343	285050	213464	446540	444961	317721	0.0304801	0.5448602
sp|Q9D2G2|	4826020	4340880	3232570	9341350	6408790	6879580	0.029126	0.5479286
sp|Q5SVQ0|	498031	372639	475218	711899	846121	877506	0.0046939	0.5526067
sp|P61327|M	10131300	12698200	10950600	21940500	17581000	20574100	0.0041969	0.562106
sp|Q9QUG9	106004	52351.7	49712.1	128943	119923	119599	0.0452188	0.5646881
tr|Q80ZP8|Q	5530930	7408710	7696510	13456500	10585900	12451100	0.0081001	0.5654747
sp|Q6VGS5|	178376	86437.9	110469	207657	218770	234518	0.0291565	0.5677975
sp|Q9DAK9	21612400	25703600	25360100	47950800	30868400	48723400	0.0375816	0.5698182
tr|A0A338P	4010810	2401000	3833700	6057500	5821280	5871130	0.008279	0.5772148
sp|Q8R3N6|	641887	774673	439643	1155920	1185490	850367	0.0371168	0.5815579
sp|Q9JJZ6|K	1336080	1780420	1684250	2386260	NaN	3109620	0.0374069	0.5823453
tr|B1AX95|E	693082	1117730	1063260	1775590	1451660	1703310	0.0143919	0.5829099
sp|Q9EQ20|	1028300	888781	1111220	1780340	1618790	1793360	0.0010961	0.5832079
sp|P70202|L	13769700	9300380	12199100	24859300	15639600	19772300	0.048523	0.5851747
tr|Q8C1C2|	162337	123450	179934	206410	262203	326065	0.046084	0.5860499
sp|P08207|S	14414200	9211340	10072500	17167000	19917100	20381000	0.0139586	0.5864088
sp|Q9D735|	10083000	10987100	11599600	17390700	16542200	21643500	0.0095881	0.587834
tr|D3YYK0|	695769	1523160	1461810	1832500	2295850	2088680	0.0470065	0.5920414
sp|Q8C570|	8963020	5571350	8211130	14199800	12392800	11638800	0.0156428	0.5949429
sp|P35486|O	9992900	11635100	9810300	19349400	18894500	14425200	0.013418	0.5969022
tr|F6U529|F	1371940	1597020	1340950	2251260	2262720	2637460	0.003245	0.6026632
tr|E9Q9J5|E	1516720	1154190	NaN	2043410	2195200	2406680	0.0194407	0.6028879
sp|Q8R086|S	3470910	3447160	3645210	5753450	5222710	6505440	0.0036283	0.6042513
tr|A0A087W	2917160	2944850	1670620	3883240	3761240	4810210	0.037421	0.6048027
sp|P97450|A	147208000	198889000	163391000	280304000	282910000	277757000	0.0019665	0.605833
tr|Q3TCF3|	830264	863401	817127	1632770	1179260	1284990	0.0184544	0.6128337
sp|Q9D0I9|S	365883	395971	315484	640846	592055	510385	0.0076837	0.6179927
sp|Q91VW3	804590000	747073000	641270000	1405530000	1056730000	965537000	0.0444914	0.63975
sp|Q9R0P4|	28234000	37833400	47563700	57985800	68184100	51047000	0.0471184	0.6411979
sp|Q60591|	3009840	3409080	3475990	6109450	4380140	4803910	0.0290982	0.647001
sp|P01901|H	19385800	26327100	16204100	35114900	30382400	30161100	0.0296411	0.647272
sp|Q99JR8|S	594043	959480	632422	1063180	1107420	1199040	0.0323762	0.6487177
sp|P53994|R	2390150	1913760	1780200	3183990	2893160	3229060	0.0072638	0.6537688
tr|E9QNL8|	2993150	2578410	2733810	4063070	4707240	3923920	0.0056164	0.6542634
sp|Q6P8X1|	1041570	971261	1101820	1278050	1779110	1699540	0.0267449	0.6547924
sp|Q8BUK6|	523160	344509	441452	712849	622907	659359	0.0167939	0.6561632
sp|Q9CX60|	14277100	19516100	17800700	27099000	24868700	26528900	0.005946	0.6572756
tr|A0A3Q4E	9194640	11914700	10787000	18847600	14906700	14740000	0.0237263	0.6577338
tr|F8WJI3|F	4537550	4898530	5008440	6073730	8196430	7605200	0.018766	0.66031
tr|Q923F1|Q	12859000	8445670	11199800	17982500	15002500	16238700	0.0228659	0.6603419
tr|E9Q1S3|E	537163	623002	447612	903037	857513	671082	0.034504	0.6611926
sp|Q8K1M6|	1231870	1504870	1058640	2119030	1838550	1755470	0.0198472	0.6643352
sp|Q9QYB5|	4257090	3278840	4238610	5559950	5741990	6418860	0.0088375	0.6644474
sp|Q9QZE7|	6933260	6845650	7209670	11846200	8869450	10749900	0.0163157	0.6670336
tr|D3YYL1|	2448900	2088750	1532980	2731080	3058560	3292130	0.0324249	0.6684413
tr|A2AJK8|A	3760740	4741260	4063350	5384280	6529060	6760050	0.0167414	0.6729014
sp|Q3THG9|	1137460	1166040	1174100	1729170	1545960	1884980	0.004718	0.6739391
sp|P56391|C	38209800	39080600	39326700	59853400	53574100	59088100	0.0007484	0.67598
tr|G3X928|G	1135100	1507970	1399020	2023220	2097790	1832720	0.0093972	0.6789173
sp|Q6P5F9|	326357	376355	496322	566566	618729	575401	0.0240965	0.681
sp|P27048|R	11720600	8234890	11897900	16074300	13339300	16803300	0.0396373	0.6892152
sp|O35954|P	456794	442049	504501	711790	710523	613760	0.0049342	0.6892405
sp|Q64433|C	355354000	441083000	347795000	596149000	550734000	503029000	0.0137969	0.6935109
sp|P27546|M	3582980	3772300	4828870	6409450	6288060	4863840	0.0465547	0.6938049
tr|B7ZCU2|	3809580	4446840	3541130	5894780	4873210	6141790	0.0225705	0.6976761
sp|P51125|I	20193100	19510400	23545900	35716700	28831200	25971600	0.0447337	0.6987378
tr|E9Q616|E	2936290	3972140	3540610	5205930	5307780	4434490	0.0212389	0.6990166
sp|Q9CXY6|	5278000	5927780	4972660	8656640	8050650	6427990	0.0325941	0.6992974
sp|P47856|G	431964	362388	438293	590834	551069	617538	0.0048179	0.700589
sp|Q9Z2U4|	586120	506777	678681	782190	887119	843786	0.0132362	0.7049387
tr|F8WHZ9|	5427800	5472750	7090910	7916920	8749070	8683120	0.0157484	0.7097472
tr|D3Z7C0|D	3317040	3559680	2714150	3979740	5223520	4259020	0.0464334	0.7124254
tr|G5E8V9|	2745790	2020300	2568650	3710330	3130690	3445140	0.0232837	0.7130688
sp|P19973|L	331188000	351792000	410701000	489960000	552362000	491442000	0.0095777	0.7130699
sp|P42232|S	1544540	1435840	1874320	2051030	2264800	2429870	0.0212818	0.7196733
sp|Q8R3B1|	17475300	17953700	14823800	22856000	22927300	23892600	0.0032624	0.7212365
sp|Q8K2B3|	19263700	15667600	16436200	22503700	24733700	23671100	0.0068229	0.7244195
sp|Q8CAK1|	525915	493282	594750	693965	801187	730252	0.0093532	0.7252378
sp|Q9D6J6|	18849800	23980100	22974400	33199800	28523000	28157700	0.0237082	0.732131
sp|P42125|E	16286700	15679200	16665500	23542900	18317400	24563200	0.0386819	0.7321415
tr|A0A075B	1871820	1550660	1898830	2159020	2600450	2491790	0.0207331	0.7338463
sp|Q9Z2I9|S	4535380	4891400	4258800	6287810	6193990	6106950	0.0010117	0.7362292
sp|Q99JY9|	86235400	98420400	100427000	131031000	125798000	128992000	0.0020114	0.7388991
sp|P30416|F	7851610	7562540	7965090	10140900	9876860	11394300	0.0051839	0.7442759
sp|Q9Z0J0|	8384040	9058890	9308000	10739600	13337000	11794100	0.0193502	0.7457599
sp|Q9Z1K5|	1145480	896915	955011	1332290	1439650	1246370	0.0220498	0.745937
tr|E9PZ00|E	16088600	13685300	13739800	20696400	21126800	16492700	0.0423025	0.7461721
sp|P51859|H	94702300	86465500	103722000	130942000	132135000	118434000	0.0083175	0.7467407
tr|A0A2I3B	333047	306324	327360	445877	374970	472951	0.0228508	0.747204
sp|Q923D4|	83018100	75738100	78210300	111830000	113269000	90424600	0.0271441	0.7510262
sp|Q61249|I	4271600	4832610	3542840	5733850	5711180	5324450	0.0256442	0.7541707
tr|A0A1B0G	3218390	3162640	2997020	3966470	4288030	4001060	0.0014002	0.7652078
sp|Q9DCX2|	69342200	82588200	76482600	105130000	89299400	103883000	0.0215365	0.7656839
sp|Q9DCW4	31687300	30780800	36119100	46362300	43390500	38803600	0.0220666	0.766879
sp|Q02819|	12797000	14330800	11219900	16403900	17785300	15707600	0.023906	0.7685403
sp|Q61035|S	5687240	5780280	5988260	4520330	4233320	4621510	0.0007467	1.3050894
sp|P21981|T	3295870	3660920	3715340	2591530	2936670	2605770	0.0081595	1.3120444
sp|Q64378|F	9395600	10611200	8620480	7534090	6880630	7318370	0.0196922	1.3172209
sp|Q8CGF7|	7808450	6983720	7855120	5233540	5761380	6012700	0.0067052	1.3315967
sp|P26516|P	10734700	13021500	11343800	9020570	9136190	8185870	0.0173809	1.332441
sp|P27659|R	16707300	15971400	18748400	14654100	10202700	12571800	0.0380993	1.3740054
sp|Q2KHI9|	266831	288686	322182	170433	216831	250701	0.0476014	1.3757792
sp|O35129|P	14600100	16152000	15763700	11217700	11900000	10678500	0.0019246	1.3763618
tr|Q3UJQ9|	6338540	5256680	6629120	4656520	3676530	4799710	0.036076	1.3877007
sp|O35345|I	646771	NaN	779049	561854	471244	503784	0.0442792	1.3916033
tr|E9PWW9|	733911	806301	793035	546642	565381	563699	0.0006819	1.3923831
sp|P99026|P	55584700	52394300	54524100	39846900	30151600	46501600	0.0338884	1.3948752
sp|Q9Z1F9|S	10744500	8707290	8974410	6403900	7231740	6391780	0.0159125	1.4193641
sp|Q9CPU0|	27129500	26199400	30103900	19973900	20739000	18031200	0.0044857	1.4202754
sp|P28843|D	1626090	1493360	1753330	1156810	1340850	929833	0.0266081	1.4216747
sp|Q8BMS1|	5791850	7884910	6748660	5368040	4649760	4299630	0.0404906	1.4266122
sp|P10126|E	252068000	281887000	195545000	173095000	158462000	166553000	0.0397597	1.4645359
sp|Q9JKF1|I	5461320	5189720	5102690	2720100	3472230	4535460	0.0356483	1.4684972
sp|P62855|R	25730600	27698500	31524800	15811100	21630000	19958000	0.0193346	1.4800563
sp|P10922|H	12668500	14424000	12452400	8062210	10795800	7510320	0.0211317	1.499712
tr|E9Q6R3|E	3310250	3109140	3291950	2735640	1581740	2121950	0.0324958	1.508129
sp|P62897|C	132658000	125348000	127069000	71722400	102218000	79777600	0.0095573	1.5177283
sp|Q9D8E6|	16862000	19657600	19820600	13238700	10598200	13038800	0.0071673	1.5278408
sp|Q9WU84	5524010	5015300	5378350	3757640	4359360	2228680	0.0464545	1.5385804
sp|P62892|R	62749000	47600100	68877400	35674100	36819500	43268200	0.0350615	1.5482353
sp|P47962|R	18339600	22032300	21305800	11174200	14948200	13450200	0.0094514	1.5585961
sp|Q8JZN5|	1325460	1788690	1783390	1058680	772143	1043590	0.019847	1.5869096
sp|P97351|R	18775200	22448800	17874100	13314000	11027300	12416500	0.0086048	1.6077703
sp|Q9CZ30|	4969040	5385660	4841180	3114210	2285120	4013020	0.0213938	1.6144619
tr|A0A0N4S	31007500	24004200	25194900	18164700	19796000	11405800	0.0376139	1.6247172
sp|P29351|P	11859700	10387400	9444190	5477010	6098070	7872810	0.0153197	1.629549
sp|P30681|H	578995000	700961000	753186000	332730000	469558000	443522000	0.016874	1.631984
tr|I7HLV2|I	4869850	6556760	5495930	2832130	3040900	4348750	0.0308968	1.6555375
sp|O08692|	9228710	10192300	9423610	4135130	6457960	6721010	0.0116534	1.6659613
sp|Q60611|S	4299290	4327990	3688990	2679820	1820200	2846620	0.0120901	1.6764494
tr|A0A0R4J	396766	318795	398969	150886	226157	282301	0.0306013	1.6903619
tr|F6TCF9|F	2314700	2575760	2389070	1697800	1376290	1194520	0.0038064	1.7053631
tr|F8WHG5|	339031	268069	421764	208538	165445	228588	0.0419466	1.7074569
sp|Q61179|I	3304820	3372950	3681250	1426210	2170850	2445190	0.0115294	1.7144309
tr|A0A1B0G	5476780	6020620	6181220	3255150	3633470	3024400	0.0007342	1.7833738
sp|Q64523|H	3005220000	3544840000	3538120000	2467020000	1651570000	1401380000	0.0144086	1.8275788
sp|Q8K194|	7439250	9454320	8329100	6083820	3984120	3699120	0.0159527	1.8321029
tr|Q8C605|Q	5682220	4738610	3757540	2629440	3067670	1916780	0.0280034	1.8621716
sp|O09044|S	1717810	1729220	1257460	644210	660130	1186550	0.0354931	1.8886783
sp|Q9QZQ8|	22858100	31515900	33543400	17204600	14304200	13976000	0.0146173	1.9328963
sp|Q9CPT5|	1395550	1627860	NaN	848443	839796	654983	0.0086636	1.9354184
sp|P14115|R	9753540	9928790	9952530	3909860	5133370	6110840	0.0016533	1.955571
sp|Q6A068|	1483620	1917790	2059930	431813	1125330	1209740	0.0407028	1.973824
sp|P47911|R	5386010	4691550	5764380	2675260	1780720	3553870	0.0121864	1.9778073
sp|P14131|R	9693570	11214700	11298500	7366130	3228110	5609700	0.0151055	1.9875888
sp|Q9Z2D6|	8676730	9668900	13239100	5078340	5899770	4838340	0.0209105	1.9969544
sp|Q3SXD3|	10186000	7483030	7210460	5804420	2692440	3867250	0.0336549	2.0122346
sp|Q64339|I	10118300	15007300	9446290	5434450	6160510	5395610	0.0296274	2.0347693
tr|F8WJ41|F	3727490	4121010	5150140	NaN	2022990	2171170	0.026889	2.0661491
tr|A0A3B2W	8461250	5338600	8948320	3012960	3626430	4214520	0.028519	2.0958503
tr|Q6ZWZ7|	12494000	15139600	11127500	5790760	5462670	7158740	0.0062122	2.1051891
sp|Q62167|D	2301950	2898140	2746260	1147210	1450730	1170390	0.002396	2.1087192
sp|Q9D6T0|	2453790	2195400	2511100	324149	1676040	1393820	0.041238	2.109685
tr|B7ZCN0|	767141	NaN	652714	NaN	380949	289406	0.0361654	2.1180643
sp|P62751|R	22434700	27600800	35476800	13544600	13690100	12182400	0.0158651	2.1694214
sp|P62082|R	20825400	23907600	36635600	12047100	14311100	10881100	0.0408939	2.1850196
tr|A0A1L1S	12218300	16353400	17206900	8886570	5051190	6927230	0.0118984	2.1940389
tr|A0A0N4S	216789000	226420000	382702000	104784000	115791000	143391000	0.0487864	2.2691982
sp|P61358|R	38095800	36888800	31402900	14192900	19281200	12835200	0.0021452	2.2973247
sp|Q9QYB1|	1766800	2093350	2777590	947388	457254	1439370	0.0370963	2.3339353
sp|Q9WVK4	417404	NaN	398458	170928	131858	213392	0.0048014	2.370874
tr|Q3TJ22|Q	2391780	3272280	3655040	1151160	1468280	1194630	0.0090212	2.4433479
tr|Q3USX5|	788729	793692	861000	186110	301054	474304	0.0047366	2.5413441
sp|Q9ERS5|	859707	899746	832058	281729	317559	410936	0.0002598	2.5652835
tr|H3BJU7|H	211741	192975	142917	46497.9	85759.3	80549	0.0095857	2.5733884
tr|V9GXE4|	1578460	1430890	1514110	NaN	606460	561118	0.0005289	2.5828167
tr|D3YZA1|	6242340	5580780	6495020	2505420	1997250	2522020	0.0003077	2.6076795
tr|D3Z7D5|D	489984	493960	885407	246356	311084	155586	0.0498585	2.6217151
sp|P47963|R	18209400	14776000	20933500	7612670	6554210	6354630	0.0036446	2.6274334
tr|Q9CQM8|	10029800	14411300	12287400	4435910	7329960	2167600	0.0178177	2.6359909
tr|A2A547|A	13088100	9821570	12417500	3403510	4807420	4676930	0.0023786	2.7411199
tr|Z4YKB8|	5249640	4941330	5587030	1589120	2220830	NaN	0.0021521	2.7608411
sp|Q04857|C	1424450	788479	779704	357147	292224	402162	0.0400307	2.8459715
sp|O35744|C	9847170	7361190	9224500	3435380	2829910	2975920	0.0017307	2.8603246
sp|Q9WTK5	922654	2036400	1901840	449153	752180	466632	0.0432346	2.9142662
sp|Q07797|L	2215520	1792040	2006970	711662	79495.5	1216880	0.0190234	2.9952279
sp|P31725|S	75374800	62358800	46070800	18040000	24532900	17349000	0.0092874	3.0673994
tr|B1ARA3|	10005100	8698430	9409850	2325980	3798410	2730760	0.0003771	3.1748056
sp|Q9CPQ8|	8900610	15966800	9673980	1976410	4752140	4032120	0.0293627	3.2099665
sp|P43274|H	813628000	817553000	927156000	197512000	319245000	271753000	0.0003282	3.2445207
tr|A0A0R4J	4066440	3033200	5400110	983533	1571810	1292230	0.0150251	3.2487363
tr|Q921R2|Q	20396700	18988000	19139000	3981320	6856500	5695240	0.000122	3.5397984
sp|P43276|H	219693000	248798000	311853000	65604800	77228600	73739900	0.0023715	3.6031404
sp|Q6ZWN5	9270360	6620270	7749070	2022030	2314320	1850660	0.0017238	3.8208602
sp|Q8BP67|	2003030	2461860	2640740	605621	905113	328352	0.0022517	3.8636747
sp|P17710|H	3866300000	4516790000	5145680000	89646500	108918000	139655000	0.0010416	3.999997
sp|P43275|H	73746500	90855900	132771000	22097900	25807100	23961800	0.0128555	4.1378411
sp|P62754|R	9006720	17564800	16847700	3860670	4064680	2065280	0.0166735	4.3459942
sp|Q9JIK5|D	808594	1698380	1182890	191890	327405	289217	0.0212246	4.5637715
sp|Q80U78|	1265430	1018570	1267000	172680	241947	362566	0.000745	4.5690067
sp|P12970|R	3928450	4903940	4484520	1314770	81004	1503600	0.002767	4.5930294
sp|P15864|H	121347000	132930000	183407000	203830000	206120000	285445000	0.0041869	4.8881667
tr|E9Q740|E	47368800	48290400	52782000	12377700	16245600	1692190	0.0010768	4.8965463
tr|F8WI35|F	3674030000	4862970000	3696460000	597238000	989659000	760698000	0.0012861	5.2110607
sp|Q64282|I	1823110	2266050	2279350	635263	209109	229491	0.0009872	5.9304679
sp|Q8K0E8|	1525270	920524	893914	146135	232124	148922	0.0107771	6.335031
sp|P62830|R	2871570	4201900	4636960	NaN	485521	457029	0.0153423	8.2828002
tr|E9PV48|E	1055720	1233230	1092580	155557	97713.6	NaN	0.0008429	8.9009673
tr|F6ZQA3|F	716854	NaN	794582	40621.8	NaN	104445	0.0053743	10.418897
sp|E9PV24|F	2736970	2646520	1729730	171373	219128	204251	0.0025156	11.959977
tr|A8DUK4|	82630100	202570000	110204000	7574770	7645410	15334000	0.0287071	12.94108
tr|J3QQ16|J	190737	177995	141606	13178.4	8542.12	16656.4	0.000456	13.298045
sp|P05366|S	1232000	1276800	1643600	0	0	0		
sp|P14901|H	967620	3561100	2587000	0	0	0		
sp|Q00623|A	1251500	1599700	1185500	0	0	0		
sp|Q6ZWV7	3028600	4320100	2781500	0	0	0		
sp|Q8BY71|	397930	512070	497480	0	0	0		
tr|A0A0R4J	152970	197050	103700	0	0	0		
tr|F8VPT3|F	246780	160740	148180	0	0	0		
tr|G3X8T9|	262560	282960	285540	0	0	0		
tr|A0A0G2J	0	0	0	2172700	1011700	1786600		
sp|P40237|C	0	0	0	1286800	1763200	190170		
sp|P52624|U	0	0	0	1178700	1272000	485410		
sp|Q3THJ3|	0	0	0	1534200	1175300	1432300		
sp|Q3UFK8|	0	0	0	368320	725590	777490		
tr|E9PZ64|E	0	0	0	781960	756220	685090		
tr|A2AGL5|	0	0	0	472160	500880	469570		
sp|Q9Z0L8|	0	0	0	840800	367330	564470		
