# Supplementary material for: Glycolytic and lipid oxidative metabolic programs are essential for freshly-isolated regulatory T cells in mice with sepsis
Source: RSC Adv. 2020 Jun 3;10(35):21000–8. doi: 10.1039/d0ra01947j (PMC9054272; doi:10.1039/d0ra01947j)
Supplement: RA-010-D0RA01947J-s004 [file RA-010-D0RA01947J-s004.pdf]

Electronic Supplementary Material (ESI) for RSC Advances.
This journal is © The Royal Society of Chemistry 2020

Protein IDs	Unique peptides	Sequence coverage [%]	Mol. weight [kDa]	Score	iBAQ Intensity 24H-1	24H-2	24H-3	sham-1	sham-2	sham-3
tr|E9QAS4|E9QAS4_	3	2	216.37	9.9004	175510	176020	256050	188050	154650	163890
sp|A2ABS9|RHG27_	3	4.4	97.047	4.9308	195190	0	296460	0	0	0
sp|A2ADY9|DDI2_M	3	21.3	44.59	31.163	5711800	3938900	6273100	6474400	4829700	6027600
tr|R4GML3|R4GML3	2	1.1	268.75	2.7031	45641	92733	44390	129220	82976	198430
sp|A2AQ19|RTF1_M	7	11.2	80.796	23.79	2929500	3464800	2784500	3446100	3817200	3009900
sp|A2AR02|PPIG_M	2	4	88.324	3.3854	289000	376950	618430	1016300	2299300	904280
tr|E9Q8N1|E9Q8N1_	2	0.1	3716	5.44	10895	22865	23579	24896	4831	5042.5
tr|A2AWA9|RBGP1_	1	0.9	120.8	2.6878	44218	0	33759	0	66243	0
sp|A2BDX3|MOCS3_	1	2	49.374	3.0171	9889800	0	0	309790	15573000	11642000
sp|A2BE28|LAS1L_	6	12.1	89.413	19.269	1305200	755560	1285800	1352500	1384600	1452400
tr|Z4YL44|Z4YL44_	2	6.6	53.924	3.4265	1966200	413380	137330	419030	204100	197170
tr|S4R1M5|S4R1M5_	2	15.8	22.514	5.5796	1405000	1999000	2024700	19863000	2309800	2653900
sp|A3KMP2|TTC38_	7	17.2	52.223	24.689	2058500	4284100	3958600	4506300	5604700	4075800
tr|A6H5Y3|METH_	2	1.5	139.07	2.7571	76055	79628	88577	124060	114760	204610
tr|A6H630|ARMT1_	2	6.2	50.548	5.1952	372110	0	208450	365000	201390	0
tr|H3BJD4|H3BJD4_	2	23.9	12.448	3.8626	391400	0	648350	0	794630	790980
tr|E9QA15|E9QA15_	11	6.9	236.25	25.863	718660	866160	641660	853670	659330	484750
sp|B2RXR6|ANR44_	14	18.4	107.38	44.233	3697700	4041500	3715600	4198800	4008900	5158800
sp|B2RY56|RBM25_	12	18.6	99.551	73.945	8065300	8083300	9579800	11734000	15366000	11279000
sp|C0HKD9|MFA1B_	7	18.2	51.954	46.028	8802900	10097000	10222000	12249000	12579000	14656000
tr|A0A0N4SV66|A0A	1	36.5	13.66	77.7	159770000	196800000	291960000	105870000	100320000	136630000
tr|A0A338P7E3|A0A	3	20.9	18.783	27.836	2955900	2086900	2924700	6120300	5043500	5594300
sp|D3YXK2|SAFB1_	11	22.4	105.1	247.84	9004100	11674000	12886000	17527000	13580000	13880000
sp|D3Z4I3|RBM24_	4	16.9	24.822	13.406	4376300	8237100	4686700	9196100	8063400	7010000
sp|D3Z7P3|GLSK_M	6	13.9	73.963	40.432	2740900	3856800	3720000	4796600	4009400	4427100
tr|E9PV24|FIBA_M	5	8.5	87.428	17.583	2017100	2300300	1319600	173150	189850	194620
sp|E9PYH6|SET1A_	5	8.5	186.06	14.879	386160	418420	335800	228180	620530	468510
tr|E9Q1P8|I2BP2_M	5	15.6	59.291	35.983	3494300	3419800	3361900	5648800	2917700	3066300
sp|E9Q394|AKP13_	3	3.5	303.97	16.149	7429400	5628200	4454300	6929600	9348000	7788100
tr|E9Q6R4|E9Q6R4_	2	3.7	237.02	9.825	99547	26958	141730	0	493430	166640
tr|A0A286YDA2|A0A	7	8.6	73.427	32.126	4841900	6698000	6648900	10248000	8166400	6436300
tr|E9Q5K9|YTDC1_	3	4.9	85.648	6.9755	543600	810470	639120	967290	1069900	1345600
tr|E9Q6J5|BD1L1_M	2	1	327.45	3.3258	41706	0	0	0	0	0
tr|E9Q7G0|NUMA1_	33	33.8	235.63	323.31	10686000	11244000	11713000	15652000	12565000	14992000
sp|E9QAT4|SC16A_	8	5.6	254.2	28.055	332530	513080	122520	905450	738570	851280
sp|F7BJB9|MORC3_	1	2.1	106.52	7.1588	87972	422260	230980	494010	726810	259220
sp|F8VPQ2|ARI4A_	2	3.1	142.05	3.6277	105820	0	0	424940	48643	191210
sp|F8VPZ9|BICRA_	2	1.5	161.35	4.7113	157770	0	144860	62056	0	0
sp|O08529|CAN2_M	5	8.9	79.871	15.441	1949700	3633600	3089400	3253800	2554800	3664400
tr|E9Q6R3|E9Q6R3_	3	21.7	18.922	15.366	2439600	2702400	2511400	2764000	1370400	2021900
sp|O08553|DPYL2_M	18	48.6	62.277	265.97	37236000	34538000	32154000	50284000	38721000	45893000
tr|B1AQR8|B1AQR8_	3	9.1	39.907	7.1014	648050	1833000	1827200	2005100	2045000	2454200
sp|Q3THM8|Q3THM8_	5	41.8	18.211	20.285	5522500	9514100	8555300	7802900	7846500	9653700
sp|O08583|THOC4_M	7	36.5	26.94	95.057	17729000	24521000	23510000	31299000	26074000	29843000
sp|O08600|NUCG_M	3	13.6	32.19	6.6316	453940	1221400	950640	126990	293480	1306500
tr|Q3TMN1|Q3TMN1_	5	15.2	50.526	20.989	2431100	2252900	4618500	4350600	3913800	2054500
sp|O08692|NGP_MO	3	22.2	19.331	15.212	6801400	8858900	7189200	4178000	5595100	6404100
tr|Q6GT24|Q6GT24_	13	65.6	24.826	323.31	56780000	96782000	78808000	94237000	78637000	97818000
tr|D3YZ19|D3YZ19_	2	32.2	9.9693	5.8919	11654000	12665000	2656100	0	23206000	6184600
sp|O08749|DLDH_M	9	23.6	54.272	42.376	9406900	14422000	9981800	15486000	14119000	16128000
sp|O08784|TCOF_M	19	18.9	135	148.56	13650000	19933000	20515000	20520000	20216000	16982000
sp|O08795|GLU2B_M	14	25	58.792	111.87	16386000	20454000	26260000	42126000	35607000	32681000
tr|B1AZS9|B1AZS9_	3	16.2	26.391	29.127	20901000	27754000	21925000	52279000	25636000	28461000
tr|G3UZ34|G3UZ34_	31	38.9	108.34	157.8	14034000	17156000	16074000	24480000	20765000	20935000
sp|O08900|IKZF3_M	6	16.2	57.967	71.895	3531400	4445000	7223200	5453500	4551500	4980300
sp|O08915|AIP_MOU	9	34.5	37.605	49.685	20265000	13209000	16682000	19721000	20469000	23313000
sp|O08997|ATOX1_M	3	47.1	7.3384	24.936	53644000	101310000	40841000	50757000	23884000	67374000
sp|O09000|NCOA3_	7	8.2	151.57	21.152	345990	225640	402230	1146700	850170	842180
sp|O09044|SNP23_M	2	15.7	23.261	5.5733	1266000	1503000	959310	650890	571930	1130600
sp|O09061|PSB1_MC	8	34.2	26.372	120.7	20081000	34510000	29231000	38357000	27980000	31604000
sp|O09106|HDAC1_M	3	17	55.074	24.713	13125000	25387000	14411000	32583000	27278000	25082000
sp|O09130|NF2IP_M	7	29.6	45.121	20.529	3747600	5228700	6736200	4764200	4372400	5185000
sp|O09131|GSTO1_M	10	40.8	27.497	27.482	9915300	23438000	12250000	24738000	16348000	17764000
sp|O09159|MA2B1_M	3	3.8	114.65	10.309	413690	141400	145880	584980	525300	167970
tr|Q9CQM8|Q9CQM	3	21.2	18.579	8.2086	7391800	12526000	9374000	4481900	6350600	2065400
sp|O35127|C10_MOU	1	14.3	13.194	5.4148	535530	1203300	1177700	3261200	1228300	1981600
sp|O35129|PHB2_MC	12	46.5	33.296	71.02	10760000	14039000	12026000	11334000	10310000	10175000
sp|O35215|DOPD_M	6	69.5	13.077	48.129	11539000	16510000	33960000	18776000	38446000	38143000
sp|O35218|CPSF2_M	9	12.7	88.382	25.427	1442600	1592400	896420	1675500	2049300	2173100
sp|O35226|PSMD4_M	7	28.2	40.703	37.401	13438000	13270000	15290000	19771000	17711000	20684000
sp|O35286|DHX15_M	20	27	91.006	99.971	9082600	12080000	9995600	9195100	8856400	11988000
sp|O35295|PURB_M	9	47.2	33.901	43.503	30805000	37987000	30482000	45387000	46146000	52434000
sp|O35309|NMI_MO	6	22.9	35.235	21.408	8664100	10462000	9592500	13903000	7653900	8617300
sp|O35326|SRSF5_M	5	27.1	30.891	25.037	28437000	49254000	45215000	43640000	32528000	39323000
sp|O35343|IMA3_MC	3	15.5	57.922	24.749	3307500	4887400	6037700	6112000	4362100	5042400
sp|O35344|IMA4_MC	2	10	57.772	4.2533	891360	213600	475420	851460	481050	559360
sp|O35345|IMA7_MC	1	2.4	59.964	4.9577	476660	0	594330	567680	408280	480030
sp|O35350|CAN1_M	17	27.8	82.105	111.05	5500700	7496700	7651700	7876100	6887300	7922600
sp|O35368|IF13_MOU	1	2.7	46.299	2.9995	227940	296310	749700	249130	231520	278030
sp|O35381|AN32A_M	9	28.3	28.537	169.7	209940000	289110000	242880000	306370000	292690000	281570000
sp|O35405|PLD3_MC	3	6.6	54.388	9.2965	3726700	6561300	2366100	2152800	2618800	3287600
sp|O35459|ECH1_MC	6	20.2	36.118	26.664	914380	3728600	1044000	2930900	3282500	2469200
tr|D3Z597|D3Z597_	1	23.2	9.5074	3.2899	15305000	0	0	0	5094000	5946300
sp|O35551|RABE1_M	3	4.4	99.523	7.0699	78594	301680	113190	68724	118360	328340
sp|O35593|PSDE_MC	4	16.1	34.577	20.474	6738400	9734800	7773100	10695000	6864400	10310000
sp|O35601|FYB1_MC	17	36.6	90.054	117.3	24711000	25137000	21291000	35435000	34227000	34982000
sp|O35638|STAG2_M	5	6.8	141.28	18.869	121050	297200	217700	667750	502730	484060
tr|Q5SVG5|Q5SVG5_	5	14.1	101.19	42.853	2385300	2318700	1712100	1895900	2160900	1967600
tr|A0A286YCB3|A0A	1	13.8	9.8559	4.4799	0	925740	0	2856900	1504100	1617300
sp|O35654|DPOD2_M	5	13.6	51.354	18.467	1499800	1091900	1437300	1273500	636680	1875900
sp|O35685|NUDC_M	10	26.8	38.358	26.619	8808200	10240000	15447000	11036000	12941000	12022000
sp|O35691|PININ_M	13	21.2	82.435	43.966	6215700	7361500	9575800	9182400	9072100	9531300
sp|O35730|RING1_M	1	7.6	42.63	4.3945	158110	328120	305600	614750	733850	556340
sp|O35737|HNRH1_M	7	42.3	49.199	323.31	87860000	121030000	128060000	139440000	122200000	134130000
sp|O35744|CHIL3_M	9	26.9	44.458	25.604	7257200	6398200	7037300	3471000	2451800	2835600
sp|O35841|API5_MO	9	15.5	57.784	19.161	4046300	4412100	3419700	3299600	4671900	2027700
tr|A0A1B0GX27|A0A	5	15.7	43.493	33.702	4391500	5092900	3743200	6041600	6520600	6992700
sp|O35857|TIM44_M	3	8.6	51.091	8.5346	599030	985870	548670	467360	728870	1006500
sp|O35864|CSN5_MC	4	13.8	37.548	11.43	1589800	1059200	1580400	2052700	2137900	2664000
sp|O35887|CALU_M	2	18.7	37.063	49.361	17195000	11648000	18230000	27053000	24949000	28240000
sp|O35900|LSM2_MC	8	52.6	10.834	91.429	36745000	35241000	36647000	65348000	28886000	42426000
tr|A0A3Q4EGP6|A0A	1	0.8	101.36	2.7284	295700	327490	243290	484880	313170	333250
sp|O35943|FRDA_MC	8	11.6	22.924	4.4818	1122800	805870	1063900	3099700	4124200	1363200
sp|O35954|PITM1_M	6	6.4	134.94	18.245	336650	384220	384880	719170	615590	584820
sp|O35955|PSB10_M	6	30.4	29.063	70.882	22613000	35056000	30193000	41381000	32087000	34494000
tr|A0A1B0GQZ1|A0A	1	12.9	11.89	3.0539	1184100	1390300	1066900	0	661940	0
tr|Q3UR63|Q3UR63_	2	10.3	37.811	4.1127	808510	0	0	242040	1415900	389460
sp|O54734|OST48_M	7	16.3	49.027	21.025	6805800	7139900	5552300	6659300	7129400	6289100
sp|O54781|SRPK2_M	1	1.8	76.756	2.7133	16625	0	67049	141220	80123	0
tr|F6TMZ3|F6TMZ3_	2	2.8	55.189	4.0181	216100	277050	295440	500090	1378900	283010
sp|O54786|DFFA_MC	6	25.7	36.572	50.676	12142000	15270000	15523000	16392000	16533000	15647000
sp|O54824|IL16_MO	27	23.5	141.43	299	26684000	31580000	28048000	37769000	36710000	37140000
sp|O54916|REPS1_M	6	13.3	86.518	23.235	1170200	810190	1372300	853880	1252800	1006700
sp|O54941|SMCE1_M	8	36.5	46.638	43.612	7415800	5465100	7618000	11865000	10780000	11825000
tr|A0A0U1RP03|A0A	2	11.6	24.95	5.8737	3126000	352450	1973100	4211500	3126300	2806800
sp|O54962|BAF_MO	6	64	10.102	56.773	6035100	51265000	36889000	137560000	62698000	45602000
sp|O54984|ASNA_M	4	17.8	38.822	9.8061	3029400	2652700	2805800	3740100	2255400	4185500
sp|O54988|SLK_MO	2	3	141.46	3.9764	59814	224480	168050	232160	293770	203220
sp|O55013|TPPC3_M	2	10.6	20.302	4.3896	684720	2452000	1719300	630680	584060	2103000
sp|O55022|PGRC1_M	2	4.6	21.694	5.1007	4508300	1141000	2699500	1111200	2104900	0
sp|O55029|COPB2_M	13	19.8	102.45	43.231	3429600	3812800	3942500	4427600	4164800	4858000
sp|O55057|PDE6D_M	3	25.3	17.348	7.6949	2560000	3950900	2605100	3280200	3897300	4201400
sp|O55098|STK10_M	3	11.3	111.9	20.864	2852000	1496300	1745900	2365700	2169900	3170000
tr|H3BJY1|H3BJY1_	1	39.1	4.6893	4.065	229410	0	0	1108900	367070	0
tr|D3Z4L7|D3Z4L7_	1	11.7	10.904	3.943	2257200	3652300	0	2930900	2034700	0
sp|O55106|STRN_M	13	28.7	85.965	71.511	6688700	5747700	5583700	9542800	8372800	8300200
sp|O55125|NIPS1_M	1	4.9	33.363	3.3982	0	3246800	1341700	1853400	1274100	1914200
tr|Q7TMG8|Q7TMG8	2	8.9	32.898	5.5907	1646900	1533800	1411100	2284900	2798700	2845100
sp|O55131|SEPT7_M	11	25	50.549	56.854	13988000	17685000	15309000	35579000	20278000	23571000
sp|O55135|IF6_MOU	3	21.2	26.511	68.465	17818000	23340000	22739000	59182000	15517000	20233000
sp|O55142|RL35A_M	3	16.4	12.554	6.3824	6028100	6542600	6066400	5619300	7606100	4044900
sp|O55201|SPT5H_M	24	28.9	120.66	323.31	13368000	12009000	11135000	16509000	16921000	17638000
sp|O55222|ILK_MOU	5	21.3	51.373	6.0821	398000	1151900	262220	315120	0	0
tr|A0A2R8VJW3|A0A	1	10.6	19.619	5.825	0	0	0	754720	0	0
sp|O70172|PI42A_M	2	13.3	46.151	13.251	3314000	3029500	3581500	2512800	3150000	2618500
sp|O70194|EIF3D_M	8	18.6	63.988	34.842	2282500	2814100	3033300	2567900	3251900	3577500
sp|O70230|ZN143_M	3	9.4	69.04	7.5181	474910	747220	464980	1046500	746150	897280
sp|O70251|EF1B_MC	5	38.7	24.693	114.82	44360000	27657000	25460000	76029000	26995000	61401000
tr|D3YX62|D3YX62_	3	17.5	26.385	8.4492	1021800	2722500	2391600	2762000	1414900	1659600
sp|O70279|ESS2_MC	3	10.9	52.603	15.933	511980	872660	794880	0	644620	449930
tr|F7B6X4|F7B6X4_	1	5.2	35.381	2.9932	0	179760	604240	0	414790	311980
sp|O70310|NMT1_M	3	7.7	56.888	6.4911	978140	1422200	1161200	1610300	1189200	1347500
tr|S4R1E5|S4R1E5_	2	16.6	17.937	7.7877	5486200	7783600	2170700	7425200	5725000	6988000
sp|O70370|CATS_MC	4	12.4	38.474	52.783	8664600	11598000	5129100	21166000	11773000	11818000
sp|O70400|PDLI1_M	11	50.2	35.774	195.65	38542000	49070000	49674000	63028000	60027000	55943000
sp|O70435|PSA3_MC	10	43.9	28.405	126.62	51048000	53794000	27651000	61959000	33662000	49773000
tr|Q8BH40|Q8BH40_	1	6.1	29.736	3.5539	197220	0	0	945680	0	1181200
sp|O70469|DOK2_M	3	8	45.522	6.0913	1008400	1033900	546980	0	772340	0
tr|Q78ZM0|Q78ZM0	8	32.7	18.762	14.737	12849000	14720000	14110000	14666000	14366000	13201000
sp|O70591|PFD2_MC	4	32.5	16.534	111.25	12161000	13931000	23178000	18636000	16089000	15665000
sp|O88271|CFDP1_M	4	19.3	32.921	20.487	19454000	13876000	15097000	22148000	18824000	27985000
tr|A0A0R4J098|A0A	3	9	65.166	41.411	255540	342540	672700	862270	881390	876420
sp|O88342|WDR1_M	25	58.7	66.406	246.29	76345000	104070000	122490000	115470000	103670000	121420000
tr|A0A0R4J0T4|A0A	4	6.5	84.387	12.755	479100	784580	808450	388170	951570	883830
tr|A0A0R4J1C2|A0A	5	22.5	22.924	34.918	13126000	15094000	13422000	16341000	17884000	14918000
sp|O88487|DC1I2_M	7	22.1	68.393	90.969	4252400	7438800	7655400	8177700	7224300	10420000
sp|O88512|AP1G2_M	3	3.7	87.862	7.2506	408120	216970	328410	283020	371980	499570
tr|Q8VC91|Q8VC91_	5	15.6	47.844	16.407	1795500	1934700	1096500	2298700	2404200	1394300
sp|O88531|PPT1_MC	2	10.5	34.49	8.9105	2100200	2528300	2377900	3778000	2518200	3592900
tr|D3Z5G4|D3Z5G4_	1	4.8	36.515	2.9408	1377500	1325700	1369300	752830	0	0
sp|O88543|CSN3_MC	4	11.8	47.832	13.42	2097300	3082700	1737100	2348300	3037500	3173000
sp|O88544|CSN4_MC	10	34.5	46.284	40.988	6871200	6671200	5481700	7525900	7236000	8587100
tr|F6QK86|F6QK86_	3	37.7	13.462	8.1356	8252800	546210	545910	4271800	494450	693930
tr|D3YZC2|D3YZC2_	1	2.8	63.751	3.3531	0	0	0	57503	0	205680
sp|O88569|ROA2_MC	19	47.6	37.402	323.31	616540000	727470000	650030000	1009900000	830960000	886160000
sp|O88587|COMT_M	2	8.3	29.486	3.5548	1375500	796750	444090	906640	1169700	1578300
sp|O88653|LTOR3_M	2	28.2	13.553	6.5627	15992000	11785000	6845200	18059000	11977000	13349000
sp|O88665|BRD7_MC	1	2.6	73.999	3.3334	0	0	124310	199230	246140	0
sp|O88673|DGKA_M	4	6.3	82.761	11.826	1267200	1494200	1305500	1271400	1192600	341130
tr|A2AGN7|A2AGN7	16	46.8	44.669	92.845	13206000	14409000	16814000	22734000	14505000	17776000
sp|O88696|CLPP_MC	4	22.1	29.8	14.957	5082700	3716900	6291600	8271800	7346500	7216200
tr|A0A0J9YU62|A0A	6	14.7	46.527	19.904	5087300	4695600	3418200	5984500	3843900	5297200
sp|O88796|RPP30_M	1	3.4	29.473	2.7712	201690	19911	175750	192490	0	182640
sp|O88811|STAM2_M	3	8.4	57.454	9.0805	1362600	1460700	1240000	1287500	1469100	1657200
tr|A0A0R4J1D9|A0A	3	7.5	58.8	8.3284	182570	424800	275940	1102700	1407600	724890
sp|O88844|IDHC_MC	4	15.7	46.674	9.4006	732240	558410	665070	511990	1324700	1386300
sp|O88874|CCNK_M	5	7.8	61.363	8.4556	462180	432690	599350	1043100	373990	642270
sp|O88890|SH21A_M	5	53.2	13.904	23.201	15489000	12769000	15659000	18661000	13651000	17399000
sp|O88952|LIN7C_M	2	14.7	21.834	2.8768	58880	0	0	69207	0	424650
tr|A0A2R8VHE4|A0A	1	4.4	24.308	2.9163	0	0	0	1231600	689380	0
sp|O89023|TPP1_MC	2	6.2	61.341	10.416	4355500	3984800	4437000	4827400	4285000	5446600
sp|O89053|CORIA_M	23	44.3	50.989	323.31	764130000	892730000	766810000	1008400000	926110000	1156600000
tr|D3Z315|D3Z315_	6	37.4	25.37	65.176	10056000	13896000	12573000	33431000	20385000	16357000
sp|O89086|RBM3_M	2	20.9	16.604	72.946	204370000	273100000	253420000	373280000	337730000	316320000
tr|G3X8Q0|G3X8Q0_	2	5.5	80.443	37.392	9834700	8884300	10543000	16052000	12141000	12343000
sp|O89100|GRAP2_M	10	43.5	36.81	195.14	28035000	26794000	29812000	41917000	35517000	40461000
sp|O89110|CASP8_M	1	2.3	55.356	86.247	21739000	13224000	8838500	14411000	11333000	14126000
tr|B2KGR2|B2KGR2	1	6.9	19.656	4.3281	1012500	1110400	1308700	1092100	2348800	1425300
sp|P00375|DYR_MC	3	21.4	21.606	9.775	3548000	3289000	3137500	4117800	3566700	2535700
sp|P00405|COX2_M	4	4.4	25.976	3.3412	2339100	1371000	1331900	3721800	1932400	1815000
[illegible]	[illegible]	[illegible]	[illegible]	[illegible]	[illegible]	[illegible]	[illegible]	[illegible]	[illegible]	[illegible]
